# Recent Advances in Flexible Temperature Sensors: Materials, Mechanism, Fabrication, and Applications

**DOI:** 10.1002/advs.202405003

**Published:** 2024-07-29

**Authors:** Lin Liu, Yingying Dou, Junhua Wang, Yan Zhao, Wenwen Kong, Chaoyan Ma, Donglin He, Hongguang Wang, Huimin Zhang, Aimin Chang, Pengjun Zhao

**Affiliations:** ^1^ State Key Laboratory of Functional Materials and Devices for Special Environmental Conditions Xinjiang Key Laboratory of Electronic Information Materials and Devices Xinjiang Technical Institute of Physics & Chemistry CAS 40–1 South Beijing Road Urumqi 830011 China; ^2^ University of Chinese Academy of Sciences Beijing 100049 China

**Keywords:** application, flexible temperature sensors, mechanical and electrical properties, preparation process, temperature‐sensitive materials, working mechanism

## Abstract

Flexible electronics is an emerging and cutting‐edge technology which is considered as the building blocks of the next generation micro‐nano electronics. Flexible electronics integrate both active and passive functions in devices, driving rapid developments in healthcare, the Internet of Things (IoT), and industrial fields. Among them, flexible temperature sensors, which can be directly attached to human skin or curved surfaces of objects for continuous and stable temperature measurement, have attracted much attention for applications in disease prediction, health monitoring, robotic signal sensing, and curved surface temperature measurement. Preparing flexible temperature sensors with high sensitivity, fast response, wide temperature measurement interval, high flexibility, stretchability, low cost, high reliability, and stability has become a research target. This article reviewed the latest development of flexible temperature sensors and mainly discusses the sensitive materials, working mechanism, preparation process, and the applications of flexible temperature sensors. Finally, conclusions based on the latest developments, and the challenges and prospects for research in this field are presented.

## Graphical abstract

This review presents the research progress on flexible temperature sensors in terms of materials, sensing mechanisms, advanced characterization techniques, preparation processes, and applications. It provides a number of ways to improve the performance of flexible temperature sensors including mechanical and electrical properties. It provides a reference for the preparation of high‐performance flexible temperature sensors.

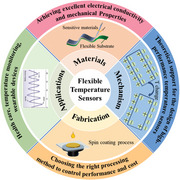

## 1 Introduction

Flexible electronics is a kind of electronic device made by micro‐nano processing of flexible materials based on their bendability, stretchability, and wearability. In recent years, major countries and regions have made plans to develop flexible electronics vigorously. According to Fortune Business Insights, the global flexible electronics market was valued at $27.1 billion in 2023, and will grow from $29.4 billion in 2024 to $71.0 billion by 2032.^[^ ^1^ ^]^ The “2023 White Paper on the Development of the Flexible Electronics Industry” presented at ICFPE 2023 and the 5th Flexible Electronics Industry Development Conference, predict that the flexible electronics market will show a long‐term growth trend.^[^ ^2^ ^]^ The rapid development and application of Artificial Intelligence and Internet of Things (IoT) technology has expanded the application areas of flexible electronics and increased its market demand. Active‐matrix organic light‐emitting diodes (AMOLED) based on flexible display electronics have been successfully used in folding mobile phones to extend their display area. In addition to applications in flexible displays, flexible energy storage,^[^ ^3^, ^4^, ^5^ ^]^ flexible generator,^[^ ^6^, ^7^ ^]^ and flexible sensing^⁠[^ ^8^, ^9^, ^10^ ^]^ began to cut a good figure.

The concept of flexible electronics dates back to the 1960s, marking the advent of pliable devices, primarily flexible sensors, that found extensive usage in industrial and medical applications.^[^ ^11^, ^12^, ^13^ ^]^ Signal sensing and transmission are the foundation of the IoT. Traditional sensors are rigid and do not have bending and stretching properties, so sensing on objects with curved surfaces is extremely limited. Flexible sensors can make up for the shortcomings of rigid sensors that are affected by shape; their excellent ability to adapt to changes in shape has led to widespread interest in fields such as healthcare, smart wearables, automotive manufacturing, and robotic tactile sensing.

Over the past decades, flexible pressure sensors have developed rapidly, resulting in a variety of microstructures to achieve high sensitivity, fast response, low power consumption, and other properties, with many experimental results.^[^ ^14^, ^15^ ^]^ As the era of IoT and 5G arrives, flexible temperature sensors become an essential part of flexible sensors other than flexible pressure sensors. Body temperature carries physiological information reflecting human health, including metabolism, mood changes, and viral or bacterial infections.^[^ ^16^, ^17^, ^18^, ^19^ ^]^ The continuous real‐time detection of temperature changes by attaching a flexible temperature sensor to human skin facilitates timely to diagnose and prevent diseases. Monitoring temperature changes in the skin near the wound caused by surgery or trauma is beneficial in informing the post‐operative recovery of the wound. In the industrial field, flexible force sensor‐based robotic arms are already able to perform actions such as grasping.^[^ ^20^ ^]^ Robotic arms with further integrated temperature sensors can execute more complex tasks. Various functional robots are emerging, developing, and applying flexible temperature sensors to grant robots tactile sensing capabilities.^[^ ^21^ ^]^

After centuries of development, traditional rigid temperature sensors have shown excellent temperature sensing performance and resistance to extreme temperature measurement environments. However, the concepts of wearable devices, electronic skin, and epidermal robots^[^ ^22^ ^]^ proposed in recent years have exposed the limitations of rigid temperature sensors. Flexible temperature sensors have emerged as the times require. It needs to have outstanding mechanical properties and excellent temperature sensing properties of traditional temperature sensors.^[^ ^23^ ^]^ The former is the distinguishing feature of flexible temperature sensors from rigid temperature sensors. Exceptional mechanical properties refer to the ability of a sensor to deform under external forces without failure. The skin at body joints can withstand more than 30% strain, and wearable devices need to withstand greater deformation.^[^ ^24^, ^25^ ^]^ However, most materials have an elastic modulus several of orders of magnitude greater than the human skin. Hence, one of the main challenges in preparing flexible temperature sensors is to achieve their flexibility. Flexible temperature sensors consist of a flexible sensitive layer, a flexible substrate layer, and a flexible electrode, which directly affect the flexibility of the sensor. Rigid materials can also be used in flexible devices to obtain deformability by optimizing the material's design and device architecture.^[^ ^26^ ^]^ On the other hand, the flexibility strategy and design of temperature sensors can't overly sacrifice their temperature measurement performance. The characteristics of high‐temperature sensitivity, fast response time, and high‐temperature accuracy ensure the accuracy, timeliness, and reliability of temperature measurement, and show excellent temperature sensing performance. Under some specific conditions, such as the patient's body temperature, the maximum temperature fluctuation is only 2 to 3 °C, and the temperature change is so small that it is almost imperceptible. Temperature sensitivity is characterized by the value of the temperature coefficient of resistance (TCR), which is the relative magnitude of the change in resistance value when the temperature changes by 1 °C. The higher the temperature coefficient of resistance, the smaller the temperature change can be detected by the temperature sensor. In addition, in flexible resistance temperature sensors, temperature fluctuations or stresses from deformation can cause resistance changes. Decoupling the output resistance signal is another challenge in preparing flexible temperature sensors. It is worth noting that tensile or compressive stresses resulting from material deformation affect the sensor's electrical output, causing temperature measurement inaccuracy or even sensor failure.^[^ ^10^, ^27^, ^28^ ^]^

**Figure 1:**
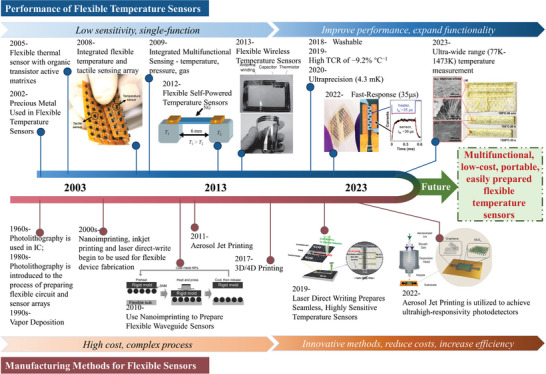
Brief timeline of the development of the performance and manufacturing methods of flexible temperature sensors. Reproduced with permission.^[^ ^35^, ^36^, ^37^, ^38^, ^39^, ^40^, ^41^, ^42^, ^43^, ^44^, ^45^, ^46^, ^47^ ^]^ Copyright 2007, Elsevier B.V. All rights reserved; Copyright 2012, Tsinghua University Press and Springer; Copyright 2013, Wiley‐VCH; Copyright 2022, American Chemical Society; Copyright 2022, IOP Publishing Ltd; Copyright 2010, Wiley‐VCH; Copyright 2019, Wiley‐VCH; Copyright 2022, Wiley‐VCH.

Compared to flexible pressure sensors, the research starting of flexible temperature sensors is quite late. A timeline of the development of flexible temperature sensor properties and preparation methods over the last two decades is presented in **Figure** **1**. After these years of development, flexible temperature sensors have made great progress in terms of sensitivity, response time, accuracy, flexibility, portability, comfort, and multi‐functionality. It is one step closer to achieving the commercial application of flexible temperature sensors. It is necessary to summarize and discuss the materials, temperature sensitivity mechanisms, preparation methods, and applications of flexible temperature sensors. During the last five years, several groups have reviewed flexible temperature sensors.^[^ ^17^, ^29^, ^30^, ^31^, ^32^, ^33^, ^34^ ^]^ It is worth noting that most of the review papers on flexible temperature sensors focus on a class of materials or a class of methods, and there are few comprehensive and complete overviews and discussions of flexible temperature sensors from materials, sensing mechanisms, preparation methods to their applications. In this review, we start by summarizing the temperature‐sensitive materials, the temperature sensing mechanism, and the flexible substrate materials to achieve the controllable performance of flexible temperature sensors, such as sensitivity, precision, and response time. Second, the preparation methods for flexible temperature sensors are classified into three categories based on the phase state of the processed materials: solid‐phase processing, liquid‐phase processing, and gas‐phase deposition. Third, application fields of flexible temperature sensors are presented. Flexible temperature sensors have outstanding potential applications in disease prediction, health monitoring, sports monitoring, and industrial production. Finally, it is concluded that flexible temperature sensors are of great significance to people's lives and industrial production, and the future development of flexible temperature sensors is also prospected.

## 2 Components of Flexible Temperature Sensor

A flexible temperature sensor consists of temperature‐sensitive material, a flexible substrate, electrodes, and an encapsulation layer. Temperature‐sensitive material is an essential component in flexible temperature sensors, which determines the mechanical and electrical properties of the material. Combining the temperature sensitivity mechanism of the material optimizes the material to obtain more excellent electrical properties. The flexible substrate supports the sensitive layer, and its mechanical properties directly affect the flexibility of the sensor. Even rigid sensitive materials can be used for flexible temperature sensors by designing structures and miniaturizing sizes on flexible substrates. Nowadays, flexible substrates can only satisfy temperature measurement applications with minor strains. Understanding the temperature measurement mechanism of materials is beneficial for preparing versatile, flexible temperature sensors. This section will review the temperature‐sensitive materials, including carbon nanomaterials, conductive polymers, metals, and other inorganic materials and common flexible substrates applied in flexible temperature sensors. The temperature sensing mechanism of different materials is also reviewed, which is conducive to obtaining actual and reliable temperature measurement and control data.

### 2.1 Temperature‐Sensitive Material

In the case of sensors, the sensitive material, the core of the sensor, converts the sensed physical signal (temperature, pressure, strain, and humidity, etc.) into an electrical signal (resistors and capacitors) that the circuit can recognize, and the electrical signal is monitored to obtain the status of the measured physical signal. In this section, temperature‐sensitive materials for flexible temperature sensors are summarized into three categories, and some novel temperature‐sensitive materials will also be introduced.

#### 2.1.1 Carbon‐Based Nanomaterials

Carbon‐based materials play a vital role in the survival and activities of living creatures in the natural world. In addition to its various electronic orbital properties of sp, sp^2^, and sp^3^ hybridization, it has various allotropes with diverse properties, including carbon nanotubes, graphene, and carbon nanofibers.^[^ ^48^ ^]^ These materials exhibit outstanding electrical and thermal conductivity, low density, extraordinarily high specific surface area, exceptional chemical stability, and, more importantly, tunable performance as required.^[^ ^49^ ^]^ Due to these excellent properties, carbon nanomaterials have been applied in many fields, such as energy and advanced electronics,^[^ ^48^, ^50^, ^51^ ^]^ especially in the field of flexible electronic devices, including flexible temperature sensors,^[^ ^52^, ^53^, ^54^, ^55^, ^56^, ^57^, ^58^, ^59^, ^60^, ^61^, ^62^, ^63^, ^64^, ^65^ ^]^ flexible gas sensors^[^ ^66^ ^]^ and flexible pressure sensors.^[^ ^54^, ^55^, ^57^, ^58^ ^]^ Using carbon nanomaterials as a temperature‐sensitive material for flexible temperature sensors has the following advantages;^[^ ^31^ ^]^ the high conductivity of carbon nanomaterials benefits the transport of carriers and back‐end circuit integration of the device.^[^ ^54^, ^67^ ^]^ Second, excellent mechanical properties. When stretching or bending the device, carbon nanomaterials still maintain good electrical connections and stable mechanical properties. Carbon nanomaterials are tightly coupled with polymer materials to form stable conductive networks through physical interaction (helical yarns) or chemical bonding (hydrogen bonding).^[^ ^23^, ^57^ ^]^ Third, carbon nanomaterials have been achieved in large‐area preparations, and raw materials are easily available.^[^ ^68^ ^]^

**Figure 2:**
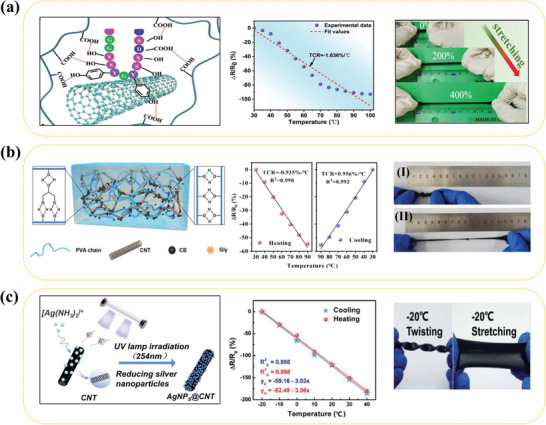
Structure, electrical properties and stretchability of CNTs modified temperature sensors by a) sericin, Reproduced with permission.^[^ ^57^ ^]^ Copyright 2021, Wiley‐VCH, b) carbon black, Reproduced with permission.^[^ ^54^ ^]^ Copyright 2020, American Chemical Society, and c) AgNPs. Reproduced with permission.^[^ ^69^ ^]^ Copyright 2022, The Royal Society of Chemistry.

Carbon nanotube (CNT), a 1D material in the shape of a hollow cylinder with a high aspect ratio, is formed by the curling of graphene. Its electrical conductivity depends on the properties of graphene and the way it is being curled. The unique high aspect ratio structure of CNTs facilitates the construction of conductive pathways and enhances carrier transport.^[^ ^55^ ^]^ Carbon nanotubes with negative temperature coefficient resistance (NTCR) properties are usually used as temperature‐sensitive materials, and their resistance decreases with increasing temperature.^[^ ^54^, ^57^, ^69^ ^]^ Typically, CNTs are dispersed in polymers to form stable carrier transport networks. Due to Van der Waals forces and strong Π‐Π interactions, CNTs are difficult to disperse uniformly and homogeneously in polymer solutions, which is detrimental to the electrical and mechanical properties of synthetic materials. To optimize the dispersion of CNTs, Lin et al. proposed adsorbing long‐chain molecular sericin onto the surface of CNTs, forming modified hydrophilic sericin carbon nanotubes (SSCNTs).^[^ ^57^ ^]^ The SSCNTs are dispersed in carboxylic styrene butadiene rubber (XSBR) to form the active material of XSBR/SSCNTs. Sericin plays two roles: on the one hand, it improves the hydrophilic properties of CNT, which helps to form a well‐dispersed CNT solution. On the other hand, the hydrogen bonds formed between the hydroxyl, amino, and carboxyl groups of SSCNT and the carboxy XSBR facilitated the formation of a stable conductive network, and sericin increases the density of the cross‐linked network. The tensile strength, Young's modulus, and toughness of XSBR/SSCNT increase with increasing SSCNT content, which corroborates the second roles of the sericin. It shows outstanding flexibility and mechanical strength – it is stretched to 400% of its initial length without cracks and holds up to 2 kg of objects without deformation (**Figure** **2**a) because of high‐density hydrogen bonds. In addition to its excellent performance in terms of mechanical flexibility, it also has highly accurate temperature sensing from 30 to 100 °C. Moreover, it shows excellent repeatability and stability with less than 5% change in resistance from 40 to 80 °C for ten cycles. The silver ammonium ions adsorbed on the surface of CNTs were reduced by ultraviolet (UV) light to obtain Ag nanoparticle modified CNTs (AgNPs@CNTs), which carry a low negative charge so that they exhibit strong electrostatic repulsion to avoid agglomeration, together with the superhydrophilicity that makes the AgNPs@CNTs well dispersed.^[^ ^69^ ^]^ Besides, the electrical properties of AgNPs@CNTs are improved, its electrical conductivity is increased three times, and its TCR is up to −3.08% °C^−1^. The order of magnitude increase of TCR is a result of the synergistic effect of the thermally excited leaps of the electrons in the AgNPs, the expansion of the substrate to reduce the space of the conductive network, and the excellent thermal conductivity of the CNTs. AgNPs@CNTs as a supportive backbone for the sensing materials, and with its content increases, the mechanical properties are improved, with a maximum tensile deformation of up to 260% (Figure 2c). The synergistic effect of the binary filler system formed by the introduction of other conductive fillers into CNTs not only aids in the dispersion of the CNTs but also facilitates the improvement of the electrical and mechanical properties. The synergistic interaction between metal nanoparticles and carbon nanotubes forms high TCR temperature sensitive materials, the synergistic interaction (hybridization) between CNTs and carbon black (CB) in favor of improving the mechanical properties of carbon nanotubes has been demonstrated and it improves carbon nanotube dispersion and enhances the interfacial interactions of polymer networks, which in turn improves the mechanical properties of CNTs.^[^ ^70^ ^]^ Gu et al. integrated carbon nanotubes (CNTs) and carbon black (CB) into a poly(vinyl alcohol)/glycerol (PVA/Gly) organo‐hydrogel and prepared a hydrogel sensor. This sensor has a high flexibility with an elongation at break up to 640% and anti‐freezing and moisturizing at −20 °C. In the range of 30–90 °C, it exhibits high‐temperature measurement linearity (>98%) and high sensitivity (−0.935% °C^−1^) (Figure 2b).^[^ ^54^ ^]^

In order to solve the signal interference caused by different sensitive materials in multifunctional sensors and to obtain accurate sensing signals, Li et al.^[^ ^56^ ^]^ prepared a flexible sensor based on an all‐carbon conductive medium, carbon nanowire coil/carbon nanotubes (CNCs‐CNTs) flexible sensor. Carbon nanocoils have a unique 3D helical morphology making the network structure highly elastic to ensure large deformation^[^ ^71^, ^72^ ^]^ The electron hopping frequency of carbon nanowire coils (CNCs) and carbon nanotubes (CNTs) increases with temperature, giving the sensor a low resistance. Together with the fact that the microcracks in CNCs‐CNTs are affected by the thermal expansion and contraction of the polymer substrate gives the sensor a high TCR.

CNTs are a promising temperature sensitive material due to their high conductivity and excellent mechanical properties. The problem of its poor dispersion in solution, which affects device preparation, can be solved by using chemical interaction, electrostatic interaction, and synergistic interaction with other materials, a process that is accompanied by an increase in both sensor sensitivity and mechanical properties.

Graphene, a 2D material with a single layer of carbon atoms,^[^ ^66^ ^]^ is a suitable zero‐bandgap semiconductor having no charge carriers at the fermi level.^[^ ^60^ ^]^ Meanwhile, its performances are adjustable and optimized by various modifications. Graphene has several derivatives, all of which have exciting properties.^[^ ^73^ ^]^ Graphene's s‐ and p‐orbitals form σ‐bonds and delocalized Π‐bonds. The abundance of delocalized electrons contributes to the outstanding electrical properties of graphene. Concentration of electrons and holes of up to 10^13^ cm^−2^ and a mobility of 200000 cm^2^ V^−1^ s^−1^. Combined with the large specific surface area of graphene and the abundance of reactive sites on its surface, these reactive sites can be used to build new structures and new functionalities through hydrophobic reactions, dipole‐dipole interactions, and the reaction of Π‐Π stacking with functional groups. It makes graphene a sensitive material for the detection of external stimuli.^[^ ^68^, ^74^ ^]^ Graphene has remarkable mechanical properties due to Π‐bond stacking.

The resistance of graphene at a given temperature conforms to the equation: R(T) = R(0)‐(h/e^2^)(4TV_0_/hv^2^E_f_τ_0_) where h is the Planck constant, e is the charge of an electron, v is the velocity, E_f_ is the Fermi energy, τ_0_ is the backscattering rate from atomically sharp defects in graphene lattice and V_0_ is the characteristic interaction constant.^[^ ^75^, ^76^ ^]^ Thus, heat conduction is achieved by both phonons and electrons in graphene. As the temperature changes, the temperature resistance effect of graphene shows a completely different trend because of the metallic^[^ ^61^, ^77^, ^78^ ^]^ and semiconducting^[^ ^60^, ^63^ ^]^ nature of graphene. Whether graphene exhibits metallic or semiconducting properties depends on whether its zero bandgap is altered or not.^[^ ^64^ ^]^ Graphene can be used as a temperature‐sensitive material for flexible temperature sensors due to its temperature‐sensitive properties and excellent mechanical properties.^[^ ^60^, ^63^, ^79^, ^80^, ^81^, ^82^, ^83^ ^]^

Similar to carbon nanotubes, although graphene has temperature‐sensitive properties, its sensitivity is not high enough to meet the demand for applications in medical and healthcare fields.^[^ ^62^, ^64^ ^]^ To improve the sensitivity of graphene flexible temperature sensors, Chen et al.^[^ ^61^, ^77^ ^]^ proposed a flexible temperature sensor with a sandwich structure consisting of a PDMS substrate, a graphene layer as active material and a PDMS encapsulation layer. The surface wrinkling of the graphene layer and the excellent mechanical properties of PDMS (Young's modulus of 3.7 MPa and tensile strain of more than 200%) ensure the flexibility of the sensor.^[^ ^77^ ^]^ The sensitivity of the graphene/PDMS sensor is as high as 5.203% °C^−1^, which is higher than most existing flexible temperature sensors, and is 40, 8, 5, and 3 times higher than that of laser‐carbonated material,^[^ ^84^ ^]^ reduced graphene oxide,^[^ ^64^ ^]^ carbon black and CNT temperature‐sensitive materials,^[^ ^54^ ^]^ and carbon nanofibers,^[^ ^85^, ^86^ ^]^ respectively. The reason for obtaining highly sensitive temperature sensors Is perhaps the formation of pores from the pore formation of the porous structure of the sensing material by the volatilization of solvents during the solidification process. A higher proportion of graphene was doped to obtain a denser graphene‐PDMS sensor, which has only half the temperature sensitivity of the porous structure sensor.^[^ ^61^ ^]^ The strategy of tuning the temperature sensitivity of the sensor by adjusting the number and structure of porosity or introducing microcracks has been demonstrated (**Table** **1**).^[^ ^68^, ^69^ ^]^

**Table 1:** Performance parameters of flexible temperature sensors based on carbon nanomaterials and conductive polymer materials.

Materials	Temperature measurement range	Sensitivity	Response/Recovery time	Accuracy	Flexible	References
PVA/Gly/CB/CNT	30–90 °C	0.935% °C^−1^	–	–	75%	[54]
graphene nanoribbon	30–80 °C	1.27% K^−1^	0.5 s	0.2 °C	flexible	[63]
carbonized PI	−10–60 °C	0.142% °C^−1^	–	–	flexible	[84]
PEDOT:PSS/CNT/WPU	25–45 °C	31uV K^−1^	0.7 s/24 s	0.3 K	bent to half of the original length	[55]
CCNT/PMIA	30–90 °C	0.95 °C^−1^	–	–	bending radius of 2 cm	[58]
CNT/PEDOT: PSS	30–80 °C	0.64% °C^−1^	2.5 s/4.8 s	–	bendable	[87]
rGO/LIG	25–45 °C	1.56% °C^−1^	13.5 s/22.6 s	0.2 °C	flexible	[88]
CNCs/CNTs	7–400 K	1.88% K^−1^	–	–	100%	[56]
Carbon Nanofiber	30–55 °C	1.52% °C^−1^	1.2 s/7.9 s	–	50%	[85]
AgNPs/CNT	−20 −40 °C	2.99% °C^−1^	–	0.1 °C	261%	[69]
XSBR/SSCNT	30–100 °C	1.636% °C^−1^	–	–	bendable and stretchable	[57]
rGO	20–100 °C	2.869%kΩ °C^−1^	1.3 s/5.5 s	–	flexible	[60]
MWCNT‐based	20–100 °C	0.064%kΩ °C^−1^	1.4 s/5.2 s	–	flexible	[60]
graphene/PDMS	30–70 °C	5.203% °C^−1^	22.4 s/31.0 s	–	flexible	[61]
LrGO	30–100 °C	0.37% °C^−1^	0.196 s/9.7 s	–	flexible	[62]
Carbonized Silk NFs	35–63 °C	1.75% °C^−1^	13.12 s	–	bending radius of 21 mm	[86]
PDMS/graphene	−40–300 °C	4.87% °C^−1^	–	0.5 °C	flexible	[77]
R‐GO/P(VDF‐TrFE)	30–80 °C	–	–	0.1 °C		[89]
Graphite/PEO/PVDF	25–42 °C	0.935% °C^−1^	26 s	0.1 °C	bending radius of 22 mm	[90]
PEDOT:PSS/PDMS	30–55 °C	4.2% K^−1^	–	0.2 °C	–	[91]
Silk/PEDOT:PSS	20–50 °C	0.99% °C^−1^	–	–	90° bending angle	[92]
(PEDOT:PSS)/PANI	30–180 °C	0.803% °C^−1^	20 ms	0.1 °C	withstand bending	[93]
TPU/CNFs	–	–	0.73 s	0.05 K	–	[94]
PEDOT/TPU	20–40 °C	0.95% °C^−1^	30 s/27 s	0.2 °C	140%	[95]

Carbonization of other materials can also be used to obtain temperature‐sensitive materials as the active materials of flexible temperature sensors, for example, laser carbonization of polyimide substrates^[^ ^84^ ^]^ and high‐temperature carbonization of silk^[^ ^86^ ^]^ have advantages in terms of convenient processing and outstanding biocompatibility in temperature sensing, respectively. In particular, laser carbonization enables the direct design of sensors on the substrate via CAD, simplifying the sensor preparation process and facilitating the integrated sensors on flexible circuit boards.

Carbon nanomaterials are one of the most popular flexible temperature sensitive materials at present. Although pure carbon nanomaterials, whether CNTs or graphene, have no advantage in their temperature sensitivity compared with other materials, they have great potential for applications in flexible temperature sensors. The carbon atoms are arranged in a hexagonal structure, with high electrical conductivity and excellent mechanical properties, which are favorable for carrier migration, device integration, deformation, and wearable applications. It has a multidimensional structure with 1D CNT and 2D graphene, and the high specific surface provides conditions for its modification.

#### 2.1.2 Conductive Polymer Materials

Conducting polymers are disordered materials consisting of polymer chains of different lengths containing many unevenly distributed defects.^[^ ^96^ ^]^ Charge is transported along the polymer chains and between the chains. Conductive polymers are negative temperature coefficient semiconductors. Its carrier transport mechanism is different from that of classical semiconductors because disorder leads to the localization of the charge wave function. In classical semiconductors, as the temperature increases, electrons jump from the valence band to the conduction band, leading to an increase in carrier density. However, the carrier density of conducting polymers is constant and independent of temperature.^[^ ^96^ ^]^ Increasing temperature leads to an increase in conductivity because of the increase in carrier mobility. The carrier transport mechanism of conducting polymers is consistent with variable‐range hopping conductivity. The relationship between conductivity and temperature is in accordance with $\sigma \;\left(T\right)=\sigma_{0}\;e^{-{\left(\frac{T_{0}}{T}\right)}^{\beta }}$ where σ_0_ is the original conductivity, T_0_ is the potential barrier height, and the exponent β is a constant that relies on the hopping process and the Fermi energy level. At the same time, conductive polymers, as a class of organic polymers, have better mechanical properties than inorganic materials and can be used as temperature sensing materials for flexible temperature sensors.^[^ ^97^, ^98^, ^99^, ^100^ ^]^ Common conductive polymers are shown in **Table** **2**.

**Table 2:** Properties and applications of common conductive polymer materials.

Conductive polymer materials	Characteristic	Application
Polyaniline	Good conductivity, optically transparent, great processability	Conductive coating, lithium ion battery electrodes
Polythiophene	Good conductivity, high stability, strong solubility	Organic solar cells, sensors, capacitors
Polyacrylic Acid	Adjustable electrical conductivity, good solubility, good biocompatibility	Conductive hydrogel
Polystyrene	Adjustable electrical conductivity, good solubility, good mechanical properties	Conductive films
Polyphenylene Sulfide	Resistant to high temperature and chemical corrosion	Electromagnetic shielding materials
Polystyrene Sulfinic Acid	Adjustable electrical conductivity, good solubility, good biocompatibility	Electrodes

Among conducting polymers, poly(3,4‐ethylenedioxythiophene) (PEDOT) has been used for transparent temperature sensors due to its high electrical conductivity an order of magnitude lower than that of metals, and remarkable stability under ambient conditions, as well as its transparency to visible light. Given the poor mechanical properties of PEDOT, PEDOT:PSS materials formed by electrostatic coupling conductive PEDOT and flexible nonconductive polystyrene (PSS) can be used as flexible temperature‐sensitive materials.^[^ ^95^, ^96^, ^97^, ^101^ ^]^ The conductivity of PEDOT:PSS is much poorer than that of PEDOT. To increase the conductivity of conductive polymers, conductive fillers are introduced into the polymer matrix such as CNTs,^[^ ^55^, ^87^, ^102^ ^]^ graphene,^[^ ^103^ ^]^ and other conductive polymers.^[^ ^93^ ^]^ The conductive mechanisms of conductive polymer composites are percolation and tunnel effect.^[^ ^104^, ^105^ ^]^ The conductive filler forms a 3D network structure in the matrix. The conductivity mechanism depends on the volume fraction of the filler.^[^ ^104^ ^]^

PEDOT, polyaniline, and other conductive polymers have lower temperature sensitivities ranging from −0.10 to −0.95% °C^−1^.^[^ ^95^, ^97^, ^98^ ^]^ Pre‐stretching the PEDOT:PSS sensitive layer to produce microcracks can increase the temperature sensitivity because of the deformation of the sensing layer and swelling of the substrate. The sensitivity is increased by 5 times as compared to the sensor without microcracks. The temperature sensor has a positive temperature coefficient (PTC) characteristic, the resistance decreases with increasing temperature, in contrast to the conductive polymer carrier‐variable‐range hopping transport mechanism because of the microcracks.^[^ ^91^ ^]^ Removal of some of the non‐conductive PSS with acid increases the conductivity and creates microcracks at the same time. The presence of these microcracks increases the temperature sensitivity of the sensor from 0.41 to 4.2% °C^−1^.^[^ ^91^ ^]^ Substrate materials with large thermal expansion coefficients are beneficial for obtaining highly sensitive sensors.^[^ ^61^, ^77^, ^100^ ^]^ Polyaniline hollow nanospheres/PDMS flexible temperature sensors reach a sensitivity of −8.1% °C^−1^.^[^ ^100^ ^]^ Interestingly, microcracking transforms the NTC effect of the conducting polymer material into a PTC effect, along with an exponential increase in TCR. However, the stability of the microcracks and the mechanisms affecting the sensitivity are not well understood. It involves several complex processes, the inherent NTCR, and the PTCR due to the thermal expansion of the polymer material, and the thermal expansion of the microcrack. Therefore, microcrack‐based PTCR devices should have more significant sensitivity increases.

**Figure 3:**
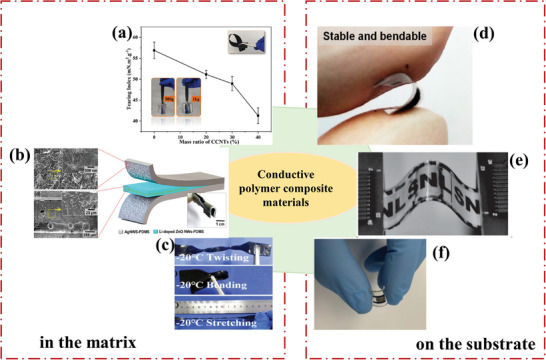
Flexible temperature sensor with conductive polymer composite as active material, sensor of left side without substrate and right side with substrate, a) a paper‐based composite sensor composed of carboxylic carbon nanotubes (CCNTs) and poly‐m‐phenyleneisophthalamide (PMIA), Reproduced with permission.^[^ ^58^ ^]^ Copyright 2021, American Chemical Society; b) Silver nanoparticles are dispersed in PDMS, Reproduced with permission.^[^ ^106^ ^]^ Copyright 2017, American Chemical Society; c) carbon nanotubes (CNTs) and carbon black (CB) are integrated into a poly(vinyl alcohol)/glycerol (PVA/Gly) organohydrogel, Reproduced with permission.^[^ ^54^ ^]^ Copyright 2020, American Chemical Society; d) PEDOT:PSS/PANI on the PI substrate, Reproduced with permission.^[^ ^93^ ^]^ Copyright 2022, American Chemical Society; e) an R‐GO/P(VDF‐TrFE) nanocomposite channel as a sensing layer, Reproduced with permission.^[^ ^89^ ^]^ Copyright 2014, Wiley‐VCH; f) a photoactive silk sericin and PEDOT:PSS on the PI, Reproduced with permission.^[^ ^92^ ^]^ Copyright 2020, American Chemical Society.

Composites are very convenient to process in the presence of polymers (**Figure** **3**). Polymer composites can be shaped and dried in a mold and then demolded to directly form flexible thin film sensors that can be bent and stretched (Figure 3a–c). Also, they can be prepared to form upper and lower structure temperature sensors on flexible substrates (Figure 3d–f). In addition, polymer fiber materials are formed by in‐situ polymerization of monomers directly on the fiber surface, which can be woven into wearable sensors. This convenient process is conducive to the preparation of sensors with good reliability and stability. Li et al. prepared an S‐type flexible temperature sensor from PEDOT‐TPU fibers to avoid the dramatic change of resistance during the deformation process. The resistance of the sensor changed less than 0.5% after 700 times of stretching, and only 1.2% when the strain reached 125%.^[^ ^95^ ^]^

In conclusion, wearable flexible temperature sensors made of polymer composites have potential applications in areas such as medical health monitoring and industrial production. Conductive polymers have the flexibility of organic materials and can adapt to the bending and deformation of flexible temperature sensors during operation. From the perspective of its material synthesis and processing, the polymer fiber material can be obtained directly through monomer polymerization and be woven into fabrics for wearable devices. However, there are still some issues that require attention. In terms of polymer temperature sensor performance, on the one hand, its stability is affected by deformation and environment; on the other hand, the temperature measurement range is too narrow to meet the needs of wearable sensors. In view of cost, the high manufacturing cost of polymer materials limits their commercialization.

#### 2.1.3 Metal and Metal Oxides

Metal, metal oxides, and other semiconductor materials were almost the earliest temperature‐sensitive materials due to fast response time, high accuracy, and wide operating temperature range.^[^ ^107^ ^]^ In metals, band theory predicts that there is no band gap between the highest and lowest energy levels, and there is no difference between occupied and unoccupied states at T = 0 K. As the temperature increases, electron motion leads to collisions between electrons, which hinders electron transport and reduces carrier mobility. Thus, metals have positive temperature coefficient properties. A different conducting mechanism from that of metals is observed in semiconductors, where there is no carrier jump between the valence and conduction bands when T = 0 K, which is equivalent to an insulator. As the temperature rises, valence electrons are excited into the conduction band. Since the carrier density of semiconductors is lower than that of metals, collisions do not affect carrier transport, so the conductivity of semiconductors increases with temperature and has NTC properties.^[^ ^96^ ^]^ The temperature dependence of the electrical resistance of metals and semiconductors allows them to be used as temperature‐sensitive materials for temperature sensors.

Metallic materials are widely used as resistance temperature detectors due to their high chemical stability, low resistance, and good linearity. Although the sensitivity (0.13–0.54% °C^−1^) of metal‐based temperature sensors is not advantageous,^[^ ^108^, ^109^, ^110^, ^111^ ^]^ metals are irreplaceable materials for temperature measurement in specific applications such as temperature calibration and high temperature detection due to their high stability.

**Figure 4:**
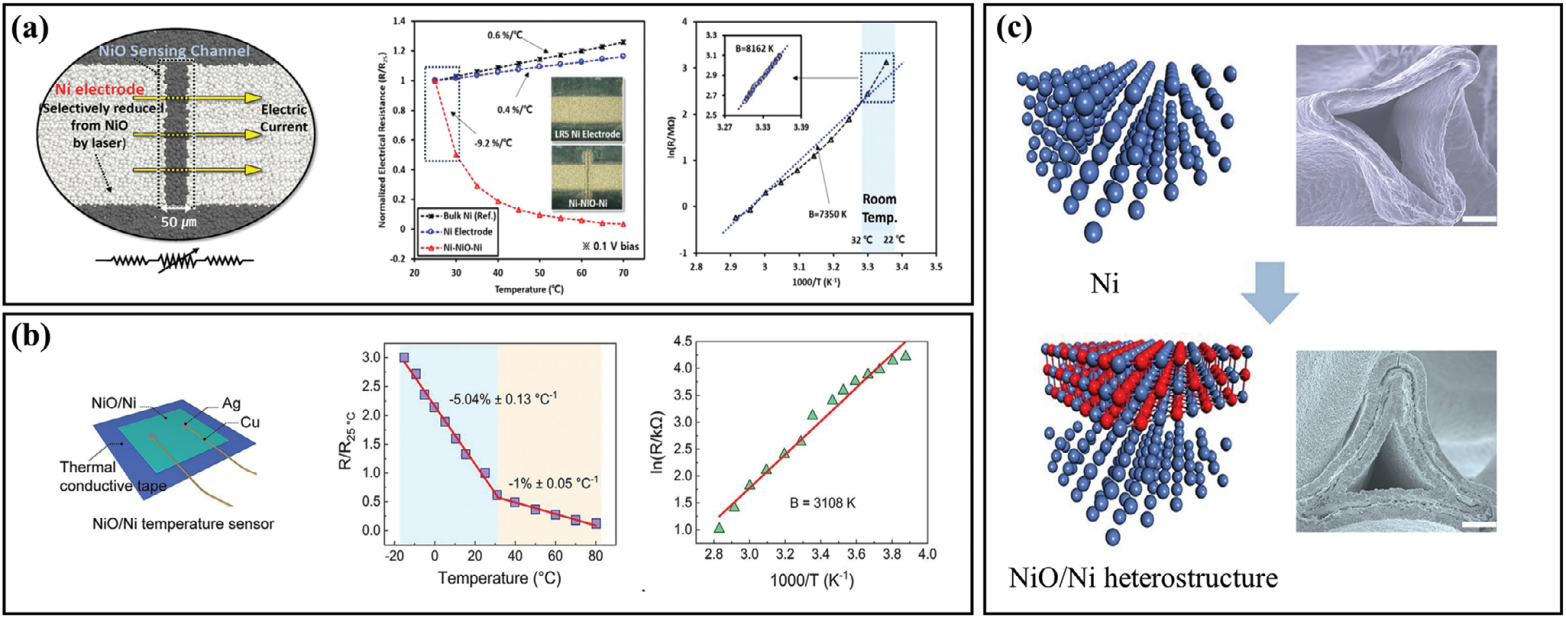
a,b) the structure and electrical properties of NiO/Ni flexible temperature sensors, Reproduced with permission.^[^ ^35^, ^67^ ^]^ Copyright 2019, Wiley‐VCH, c) Atomic structure and SEM images of the Ni and NiO framework, Reproduced with permission.^[^ ^67^ ^]^ Copyright 2021, AIP Publishing LLC.

Metal oxides provide higher sensitivity and a more comprehensive temperature range than the element metallic materials. For example, nickel oxide is a sensitive layer material for temperature sensors because of its large resistance temperature coefficient and chemical stability. Huang et al.^[^ ^28^ ^]^ prepared NiO thermistor arrays via inkjet printing with a temperature coefficient resistance of −4% °C^−1^. Unique structures, such as metal‐semiconductor‐metal (MSM) structures formed using the oxidizing properties of metals and the reducing properties of metal oxides, often have some unique properties. Laser‐induced reductive sintering reduced nickel oxide to form nickel electrodes in situ.^[^ ^35^ ^]^ The formed Ni‐NiO‐Ni seamless structure has the highest sensitivity of any previously reported NTC thermistor. In addition, the resistance temperature coefficient can be adjusted by controlling the width of the NiO channel (**Figure** **4**a). Given that the high resistivity of NiO significantly limits the carrier transfer rate and the practical application of wearable devices. Zhang et al.^[^ ^67^ ^]^ proposed a simple and controlled in situ oxidation method for the preparation of NiO/Ni heterostructure frameworks. This structure in the temperature sensor is characterized by high sensitivity, adjustability, stability, high carrier concentration, and a wide temperature range (Figure 4b). Different thicknesses of Ni metal layers are obtained by controlling the oxidation time, which is the key to achieving adjustable sensitivity (Figure 4c).

**Figure 5:**
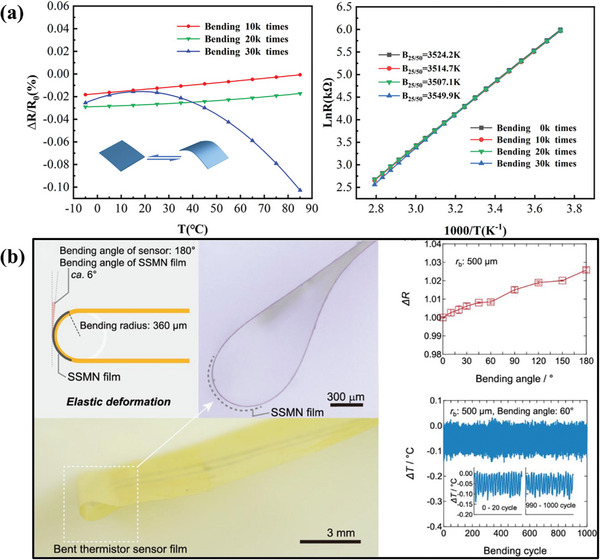
a) Resistance drift rate and B value of Mn‐Co‐Ni‐O flexible temperature sensors after bending for different times, Reproduced with permission.^[^ ^112^ ^]^ Copyright 2023, Wiley‐VCH. b) Stability and reliability testing of Sr‐doped SmMnO_3_ flexible temperature sensors, Reproduced with permission.^[^ ^36^ ^]^ Copyright 2020, American Chemical Society.

Despite the high and tunable sensitivity of this material reported in these two pieces of literature, the flexibility and stability of the sensor have yet to be evaluated, which is an important factor in achieving flexible sensor applications. In order to characterize the flexibility and reliability of flexible temperature sensors, Song et al.^[^ ^112^ ^]^ repeatedly bent and restored the all‐inorganic Mn‐Co‐Ni‐O thin films. Bending causes a change in resistance. When the sensor is bent 30 thousand times, it still provides stable temperature sensing with a resistance drift of only 0.1% (**Figure** **5**a). Flexible 3 × 3 Sr‐ and Ni‐doped perovskite SmMnO_3_ thermistor film sensor arrays were inkjet printed on PI substrates.^[^ ^36^ ^]^ These ceramic temperature sensor arrays are ultrathin, highly flexible, and featherweight. As the individual dot shape is small (diameter is 900 um), the sensors have a small bending radius (500 um). When sensors are bent repeatedly with a 500 um bending radius, the resistance varies slightly by 2.5% (Figure 5b). Song and Tomohiko's study shows that rigid metal oxide ceramics can also be an active material for flexible temperature sensors with excellent flexibility and stability. This reliability and flexibility offer a possibility for applying metal oxide‐based flexible temperature sensors. The performance parameters of metal‐based and other inorganic material‐based flexible temperature sensors are listed in **Table** **3**.

**Table 3:** Performance parameters of flexible temperature sensors based on metals and other inorganic materials.

Materials	Temperature measurement range	Sensitivity	Response/Recovery time	Accuracy	Flexible	References
Ag NCs	85–370 K	0.134% K^−1^	–	–	–	[110]
Ag NCs	85–370 K	−0.512% K^−1^	–	–	–	[110]
Ag NWs	−20–20 °C	0.33% °C^−1^	–	–	–	[108]
Ni	5–40 °C	4250 ppm °C^−1^	30 ms	–	flexible	[113]
Ni	−60–80 °C	0.44% °C^−1^	10 s	–	flexible	[109]
NiO	29–70 °C	−9.2% °C^−1^	50 ms	–	–	[35]
NiO/Ni	−15–80 °C	−5.04% °C^−1^	–	–	–	[67]
MCN film	19–130 °C	4429 K	1.0 s	–	–	[114]
Mn‐Co‐Ni‐O thin film	−5–85 °C	−3.9% °C^−1^	–	–	bending radius 10 mm	[112]
h‐BTNCs	20–45 °C	20 nA °C^−1^	24 ms	4.3 mK	–	[115]
BaTiO_3_	28–41 °C	48 mV °C^−1^	–	–	flexible	[116]
Ni/Sr‐SmMnO_3_	40–430 °C	1572 K	–	0.1 °C	bending radius 500 um	[36]
MoS_2_	30–80 °C	2% K^−1^	36 us	–	–	[37]
Ag_2_S	20–90 °C	−4.7% K^−1^	0.11/0.11 s	0.05 K	–	[117]

Metal oxide materials exhibit excellent temperature sensitivity and stability for temperature sensing. However, metal oxides are not inherently flexible or extensible compared to the carbon nanomaterials, conductive polymers, and metals mentioned above, which limits their application in flexible temperature sensors. Flexible temperature sensors formed by preparing small‐sized metal oxide ceramics or ceramic films on flexible substrates have a large bending radius. The compatibility of the sintering temperature of the ceramics with the tolerance temperature of the flexible substrate must be taken into account. In addition, metal oxide powder materials can be used directly for temperature sensing, avoiding the high temperature treatment process, compatible with low temperature processing, and also obtaining high temperature sensitivity, which provides a new idea for the preparation of high sensitivity temperature sensing.

#### 2.1.4 Other Materials

**Figure 6:**
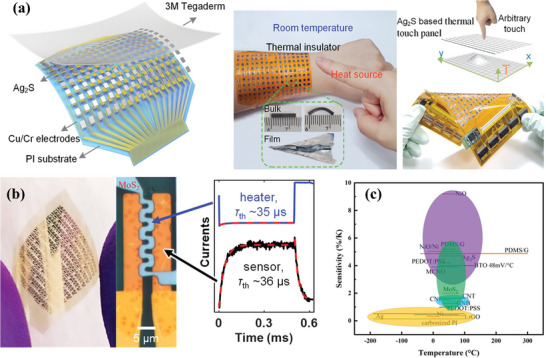
High Performance Metal Sulfide Temperature Sensor Array, a) Ag_2_S, Reproduced with permission.^[^ ^117^ ^]^ Copyright 2022, Wiley‐VCH, b) MoS_2_, Reproduced with permission.^[^ ^37^ ^]^ Copyright 2022, American Chemical Society. c) Temperature measurement ranges and sensitivity for various materials.

Transition metal sulfides (TMS), an important class of flexible inorganics, are used as excellent electrode materials for various types of electrochemical energy storage and high‐specific‐power photovoltaics^[^ ^118^, ^119^ ^]^ due to their ultrahigh optical absorption coefficients, desirable band gaps, and self‐passivated surfaces.^[^ ^120^ ^]^ Metal sulfides with negative temperature coefficient of resistance (NTCR) behavior were found as early as the 19th century. The temperature dependence of the electrical conductivity (*σ*) is the equation $\sigma \propto e^{-\frac{E}{2kT}}$, where *E* is the bandgap, and *k* is the Boltzmann constant. More importantly, metal sulfides have excellent intrinsic flexibility.^[^ ^37^, ^117^, ^121^, ^122^ ^]^ In contrast to brittle conventional inorganic semiconductors consisting of covalent and ionic bonds, TMS has metal‐like ductility due to the lower slip energy and higher solvation energy between crystal planes.^[^ ^123^ ^]^ As a fully inorganic semiconductor, TMS not only retains the large TCR (−4.7% K^−1^) of inorganic materials but also has the flexibility of organic materials (ductility: 5%–100% in metal and organic materials, 4.2% in TMSs, 0.1%–0.2% in other inorganic semiconductors), ^[^ ^117^, ^124^ ^]^ which provides an insightful strategy to achieve high‐performance flexible temperature sensors.^[^ ^37^, ^117^ ^]^ Ag_2_S is the first material found to have NTCR behavior. Recently, a high‐performance flexible temperature sensor was reported using plastic Ag_2_S with an ultrahigh temperature coefficient of resistance of −4.7% K^−1^, resolution of 0.05 K, and rapid response/recovery time of 0.11/0.11 s^[^ ^117^ ^]^ (**Figure** **6**a). Fast‐response flexible temperature sensors with atomically thin MoS_2_ can achieve real‐time thermal sensing on flexible substrates^[^ ^37^ ^]^ (Figure 6b). Therefore, flexible temperature sensors with transition metal sulfides as sensitive materials show strong strengths in temperature sensitivity, response time, and mechanical properties.

In general, the various temperature‐sensitive materials have their advantages and disadvantages. The sensitivity and temperature measurement range of the four types of temperature sensors above are shown in Figure 6c. Regarding electrical performance, metal materials have the widest temperature measurement range, followed by metal oxides. Both metal oxide and polymeric materials have high sensitivity. However, the narrow temperature range will limit the range of polymer materials that can be used. Metal sulfides and metal oxides have a fast temperature response time. Considering the requirements of flexible temperature sensors for material flexibility, carbon, polymer, and sulfide materials are all excellent and flexible. Hence, they do not require unique structures to be designed to achieve sensor flexibility. Although metals are ductile, they still need to be designed as S‐shaped patterns to achieve sufficient flexibility in flexible devices. Compared to the four materials mentioned above, the oxide is the poorest in flexibility. Currently, most of the research on flexible temperature sensors focuses on enhancing the sensitivity of flexible temperature‐sensitive materials, while neglecting the research on the flexibility and other properties of temperature sensors such as temperature measurement accuracy, response time, and repeatability. Meanwhile, there is a lack of sufficient attention to realize the application of non‐flexible and highly sensitive temperature‐sensitive materials in flexible temperature sensors. It is worth mentioning that in the near future, flexible sensors based on all‐inorganic materials will become a major research highlight and are expected to be commercialized.

### 2.2 Flexible Substrates

**Figure 7:**
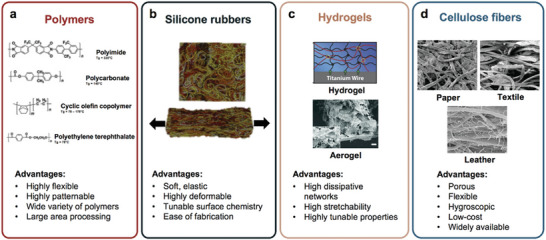
Classification of flexible substrate materials, Reproduced with permission.^[^ ^24^ ^]^ Copyright 2019, Wiley‐VCH.

The flexible substrate is one of the most important parts of flexible temperature sensors and other flexible electronic devices. It provides increased flexibility and decides the overall performance of the device. The adhesion between sensitive layers and substrates and flexibility must be considered factors in evaluating the suitability of a substrate for flexible devices. In addition, it is crucial to select the appropriate flexible substrate based on the application's transparency and gas‐permeable requirements. If the flexible temperature sensor is used on human skin, it must be gas‐permeable to avoid skin stimulation. Joo Chuan Yeo and co‐workers summarized the four categories of materials used as flexible substrates (**Figure** **7**).^[^ ^24^ ^]^

#### 2.2.1 Resinous Polymer Materials

**Figure 8:**
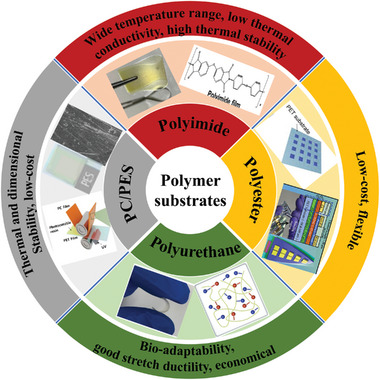
Schematic illustrations of common polymer substrates, including PET, Reproduced with permission.^[^ ^67^, ^115^ ^]^ Copyright 2021, AIP Publishing, Reproduced with permission. Copyright 2018, American Chemical Society, PI, Reproduced with permission.^[^ ^126^ ^]^ Copyright 2021, American Chemical Society, PU, Reproduced with permission.^[^ ^131^ ^]^ Copyright 2021, American Chemical Society, PES, Reproduced with permission.^[^ ^133^ ^]^ Copyright 2023, MDPI, and PC, Reproduced with permission.^[^ ^134^ ^]^ Copyright 2023, Royal Society of Chemistry.

Common polymer substrates without silicone rubber of flexible temperature sensors include polyimide (PI), polyester (PET), polycarbonate (PC), polyurethane (PU), poly(ethylene phthalate) (PEN), polyethersulfone (PES), etc (**Figure** **8**). Among them, PI is used in diverse applications due to its high dielectric voltages, excellent mechanical properties, and outstanding chemical and thermal stability.^[^ ^125^, ^126^ ^]^ Compared to other polymer materials, PI is widely used in flexible temperature sensors for high‐temperature areas due to higher glass transition temperatures.^[^ ^28^, ^36^, ^126^ ^]^ PI have poor coefficient of thermal conductivity and insulating resistivity,^[^ ^109^, ^127^ ^]^ which provide high sensitivity and rapid response time to flexible temperature sensors.^[^ ^113^ ^]^ PU has attracted wide attention owing to its bio‐adaptability and high plasticity. Various structures – PU sponge, polypyrrole‐PU, waterborne PU, and thermoplastic PU substrate provide highly sensitive, multifunction, self‐healable properties in flexible sensors.^[^ ^55^, ^128^, ^129^, ^130^, ^131^, ^132^ ^]^ When used at high temperatures, it is necessary to pay attention to the material's ignition point, especially the PU sponge. PI and PU are not suitable for a wider application because of the expensive costs. PET is more cost‐effective while ensuring excellent mechanical properties. Flexible sensors based on highly elastic PET substrates have been frequently incorporated in enormous wearable applications.^[^ ^67^, ^110^, ^115^ ^]^ These organic polymer materials, as flexible substrates, can provide sufficient flexibility, stretchability, and bendability for electronic devices. However, some problems still need to be solved urgently in practical applications. Even though PI already has a high glass transition temperature and a resistance temperature of ≈450 °C, conventional semiconductor processes must be heat‐treated at more than 1000 °C. Apart from this, organic polymer materials have a very high coefficient of thermal expansion, and their expansion is ten times that of inorganic materials, which is not favorable for preparing inorganic sensitive layers on polymer materials (**Table** **4**).

**Table 4:** Characteristics of polymer materials.^[^ ^18^ ^]^

Material	CTE(10^−6^/K)	T_g_ [°C]	Price	Color	Moisture Absorption
PI	20–30	240	expensive	orange	high
PU	20	−30–70	expensive	translucent	high
PET	60	70–160	inexpensive	clear	moderate
PC	65	150–200	inexpensive	clear	moderate
PEN	50	120	inexpensive	clear	moderate

#### 2.2.2 Rubber‐Based Polymer Materials

**Figure 9:**
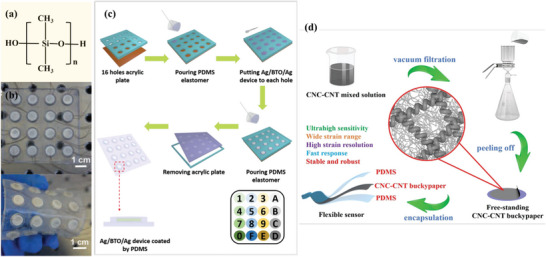
Application of PDMS in flexible/wearable devices. a) The structure of PDMS, fabrication process of the b,c) BTO ceramic array, Reproduced with permission.^[^ ^116^ ^]^ Copyright 2019, Wiley‐VCH, and d) CNC‐CNT buckypaper^[^ ^56^ ^]^ based on PDMS substrate and encapsulated layer, Reproduced with permission. Copyright 2021, Elsevier B.V.

Rubber is a polymer material that is more flexible and extensible than resins or plastics. Polydimethylsiloxane (PDMS) is a typical rubber material^[^ ^61^, ^78^, ^87^ ^]^ with good thermal stability and electrical insulation, high chemical stability, and excellent stretchable mechanical properties (Young's modulus of 0.5 to 4 MPa, elastic limit of 200%), making it one of the most commonly used flexible substrate materials.^[^ ^61^, ^135^ ^]^ Its structure is shown in **Figure** **9**a. PDMS has been widely used in wearable devices (Figure 9) in wearable devices because of transparency, controlled modification, waterproofing, nontoxicity, biocompatibility, excellent stretchability, and easy use. Ecoflex^TM^ (Smooth‐On, USA) is a rubber material with variable Shore hardnesses for commercial applications.^[^ ^136^ ^]^ Compared with PDMS, Ecoflex exhibits a lower Young's modulus (0.05–0.1 MPa, proximity to human skin, ten times PDMS) and higher stretch limit(≈1000%).^[^ ^135^ ^]^ Considering that temperature sensors for e‐skin need to have mechanical flexibility similar to human skin and high stretch properties, Ecoflex becomes an ideal candidate due to its strong elasticity and excellent ability to establish intimate contact with the skin surface.^[^ ^137^ ^]^

Generally speaking, silicone rubber materials have excellent flexibility and stretchability and can be used as a substrate for wearable materials to make perfect contact with the surface being tested. However, similar to resins, they also face the difficulty of not tolerating high temperatures (operating temperatures of less than 400 °C).^[^ ^138^ ^]^

#### 2.2.3 Hydrogels

**Figure 10:**
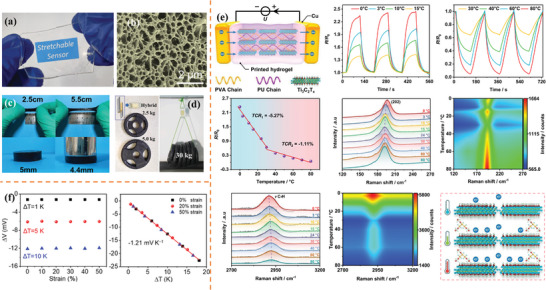
Properties and applications of hydrogels. a) Optical pattern^[^ ^139^ ^]^ and b) scanning electron microscope microstructure^[^ ^145^ ^]^ of hydrogels, Reproduced with permission. Copyright 2021, The Royal Society of Chemistry and Copyright 2022, Springer Nature. Hydrogel with excellent c) stretchability^[^ ^150^ ^]^ and d) adhesion properties, Reproduced with permission.^[^ ^143^ ^]^ Copyright 2021, Wiley‐VCH and Copyright 2021, Elsevier Ltd. e,f) Temperature sensing properties of hydrogels, Reproduced with permission.^[^ ^145^, ^146^ ^]^ Copyright 2022, Springer Nature and Copyright 2022, American Chemical Society.

Hydrogels, hydrophilic cross‐linked polymer networks dispersed in water,^[^ ^139^ ^]^ are a 3D and hydrophilic material with ultrahigh elasticity, stretchability, and toughness^[^ ^140^ ^]^ (**Figure** **10**a–d). It has excellent mechanical properties and ionic conductivity properties analogous to human skin. The hydrogel has a higher elastic stretch limits and a lower elastic modulus (1100%, 3.6 kPa) than PDMS (220%, 350 kPa) and Ecoflex (720%, 153 kPa).^[^ ^141^ ^]^ Unlike PDMS, which uses different cross‐linking agents to change the modulus of elasticity, the polymer network structure of hydrogel is permeated by water molecules, which can dissipate a large amount of mechanical energy to keep the material flexible and stretchable under external forces. The combination of hydrogel matrix and conductive filler not only enhances the conductivity of the hydrogel, but also facilitates the mechanical properties. In addition, hydrogels exhibit resistance to freezing, drying and biocompatibility.^[^ ^142^ ^]^ Hence, hydrogels are promising candidates for fabricating flexible wearable sensors for emerging fields, especially flexible pressure and strain sensors.^[^ ^143^, ^144^ ^]^ More interestingly, hydrogels also present temperature sensation abilities.^[^ ^139^, ^145^, ^146^ ^]^ PVA is one of the hydrogel materials that have been widely used in the last decade, and flexible sensors based on PVA hydrogels have enhanced mechanical properties, close conformability to the human body, and biocompatibility.^[^ ^142^ ^]^ In addition to PVA, these materials, poly(N‐isopropylacrylamide),^[^ ^147^ ^]^ poly(N‐isopropylacrylamide)‐co‐(Npropylacrylamide),^[^ ^148^ ^]^ 2‐(2′‐methoxyethoxy) ethyl methacrylate/poly (ethylene glycol) methyl ether methacrylate (MEO2MA/PEGMA),^[^ ^149^ ^]^ polyacrylamide (PAM),^[^ ^139^, ^146^ ^]^ are also popular hydrogel materials. Liu et al. fabricated MXenes bonded PU/PVA hydrogel with a high temperature coefficient of resistance (−5.27% °C^−1^ at the temperature from 0 to 30 °C, −1.11% °C^−1^ at the temperature from 30 to 80 °C) in the Figure 10e.^[^ ^145^ ^]^ Fu et al. fabricated a stretchable, self‐powered hydrogel material as temperature‐pressure dual‐sensing ionic skins. As shown in Figure 10f, it enables dual parametric measurement of temperature and strain, ensuring high temperature sensitivity without any interference of the output signals.^[^ ^146^ ^]^

Hydrogels cannot be applied in low temperature (T < 0 °C) and high temperature environments due to the tendency of water to freeze at low temperatures and volatilize at high temperatures. The introduction of glycerol not only makes the PVA hydrogel resistant to freezing and moisture, but also possesses self‐healing, antimicrobial properties, and thermoplasticity.^[^ ^54^, ^69^ ^]^ PVA/borax/gly/ silver nanoparticle hydrogel with high TCR (−2.99% °C^−1^) and low elastic modulus (189 kPa) in the range of −20 to 40 °C is promising to be applied in human wearable flexible temperature sensors.^[^ ^69^ ^]^ To further extend the operating temperature range of the hydrogel, the addition of the inorganic salt LiBr to the hydrogel resulted in a flexible temperature sensor with a high TCR (2.54% °C^−1^), a wide temperature range (−78.5 to 97 °C), and an ultra‐high stretchability (625% strain).^[^ ^54^ ^]^

Hydrogels have an ultra‐high tensile limit superior to that of PDMS, and their excellent mechanical properties have attracted the attention of many researchers. Hydrogels' instability at higher temperatures, freezing temperatures, and narrow working temperature ranges are problems to be solved.

#### 2.2.4 Cellulose Fibers

**Figure 11:**
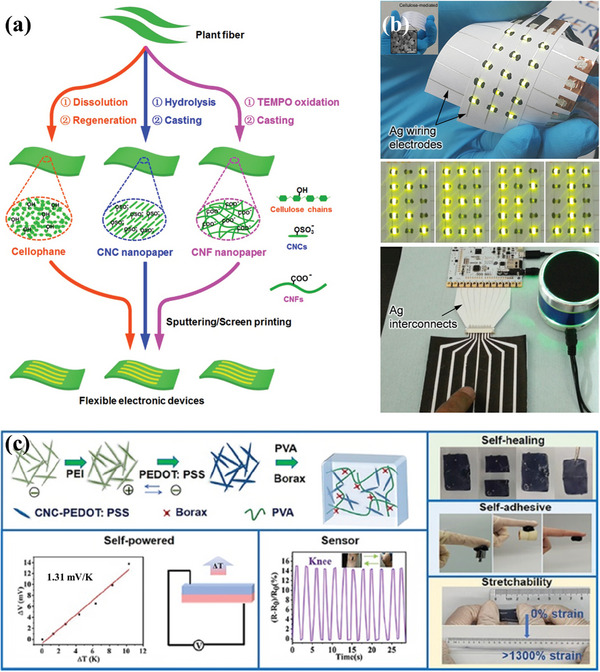
a) Preparations of cellulose films. Reproduced with permission.^[^ ^152^ ^]^ Copyright 2020, American Chemical Society. b) Photographs of cellulose paper‐based flexible devices.^[^ ^154^ ^]^ Copyright 2016, American Chemical Society. c) Manufacturing process and properties of CNC/PEDOT:PSS/PVA hydrogels,^[^ ^156^ ^]^ Copyright 2023, American Chemical Society.

Nowadays, the topic of environmental protection has been responded by countries all over the world. Due to the difficulty of degradation of plastic and rubber materials to produce white pollution, studying and preparing easily degradable and non‐polluting flexible materials to replace plastic and rubber is an important direction. Cellulose is a renewable and degradable natural polymer. About the dissolution strategy of cellulose nanomaterials in flexible sensors, Liu et al. have done made a very detailed description and discussion.^[^ ^151^ ^]^ **Figure** **11**b shows cellulose modified with different groups directly from plant fibers, including cellulose chains, cellulose nanocrystals (CNCs), and cellulose nanofibers (CNFs).^[^ ^152^ ^]^ These groups form a strong network structure within cellulose, which makes it insoluble and processable.^[^ ^151^ ^]^ Owing to the good stability, stretching, and biocompatibility, cellulose has been used for a wide range of applications in flexible electronic devices and implantable medical products, among others.

Cellulose‐based paper is a substrate material suitable for a wide range of processing methods, such as roll‐to‐roll processing, screen printing, inkjet printing, etc., which makes it very attractive in the field of flexible printed electronics. In addition, the cellulose‐based paper substrate has superb durability with an operating life of up to 10 years;^[^ ^153^ ^]^ the electrodes on the cellulose‐based paper substrate exhibit good mechanical stability with up to 10000 bending cycles (Figure 11b).^[^ ^154^, ^155^ ^]^ The composite properties made by dissolving cellulose and mixing it with other materials obtained very good properties. The composite material made by dissolving cellulose and mixing it with other materials obtained very good properties.^[^ ^151^, ^156^ ^]^ The CNC/PEDOT:PSS/PVA hydrogel with better cyclic stability, self‐healing, self‐adhesion, and excellent stretchability is demonstrated in Figure 11c.

#### 2.2.5 Other Substrate Materials

All four materials have excellent flexibility but are not resistant to high temperatures. They are not suitable for processing temperatures or working temperatures exceeding 400 °C. Mica is a well‐known layered natural insulating material.^[^ ^66^ ^]^ Due to the layered structure of mica and the ease of stripping, it is possible to strip mica into substrates of different thicknesses. Within a specific thickness range, the smaller the mica thickness, the smaller the bending radius and the better the bendability. It is coupled with the fact that mica is a fully inorganic material that can withstand extremely high temperatures (>1000 °C). Therefore, using mica as a substrate material has recently attracted widespread attention in flexible electronics.^[^ ^112^, ^157^ ^]^

Above mentioned the former four types of substrate materials, resin substrate materials have a high degree of flexibility, and can be bent, but its modulus of elasticity is too large to match the high elasticity of human skin. Rubber‐based materials have a modulus of elasticity similar to that of human skin are more stable, and are commonly used as substrate and encapsulation materials. Hydrogels have ultra‐high stretch limits and ultra‐low modulus of elasticity superior to PDMS, but they are unstable due to water evaporation and freezing. Cellulose materials are low cost and environmentally friendly. Although the mechanical properties of these materials can already meet or even exceed those of skin, they almost all face a common problem of non‐resistance to high and low temperatures. Mica is a class of purely inorganic materials. A single layer of mica has excellent flexibility and can match high‐temperature processing techniques. High tensile limit, low elastic modulus, high stability, low‐cost, easy processing, and high temperature resistant substrate materials have been formed. Suitable substrate materials can be selected according to the processing materials and processes.

### 2.3 Mechanisms of Temperature Sensing

**Figure 12:**
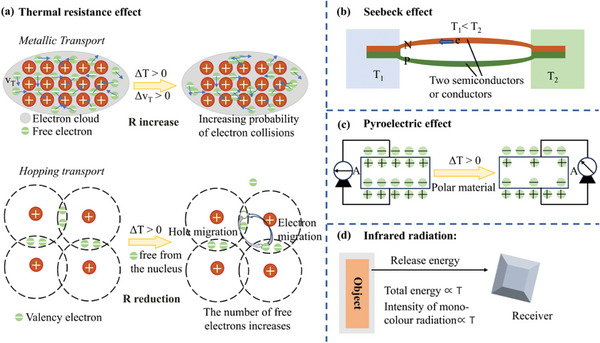
Four types of temperature sensing mechanisms.

Types of temperature sensors that have been previously reported are resistance, thermistor, thermocouple, and infrared, etc., and their sensing mechanisms include the thermal resistance effect, Seebeck effect, pyroelectric effect, and infrared radiation.^[^ ^78^, ^87^ ^]^ The mechanism of these four temperature sensing is shown in **Figure** **12**.

#### 2.3.1 Thermal Resistance Effect

**Figure 13:**
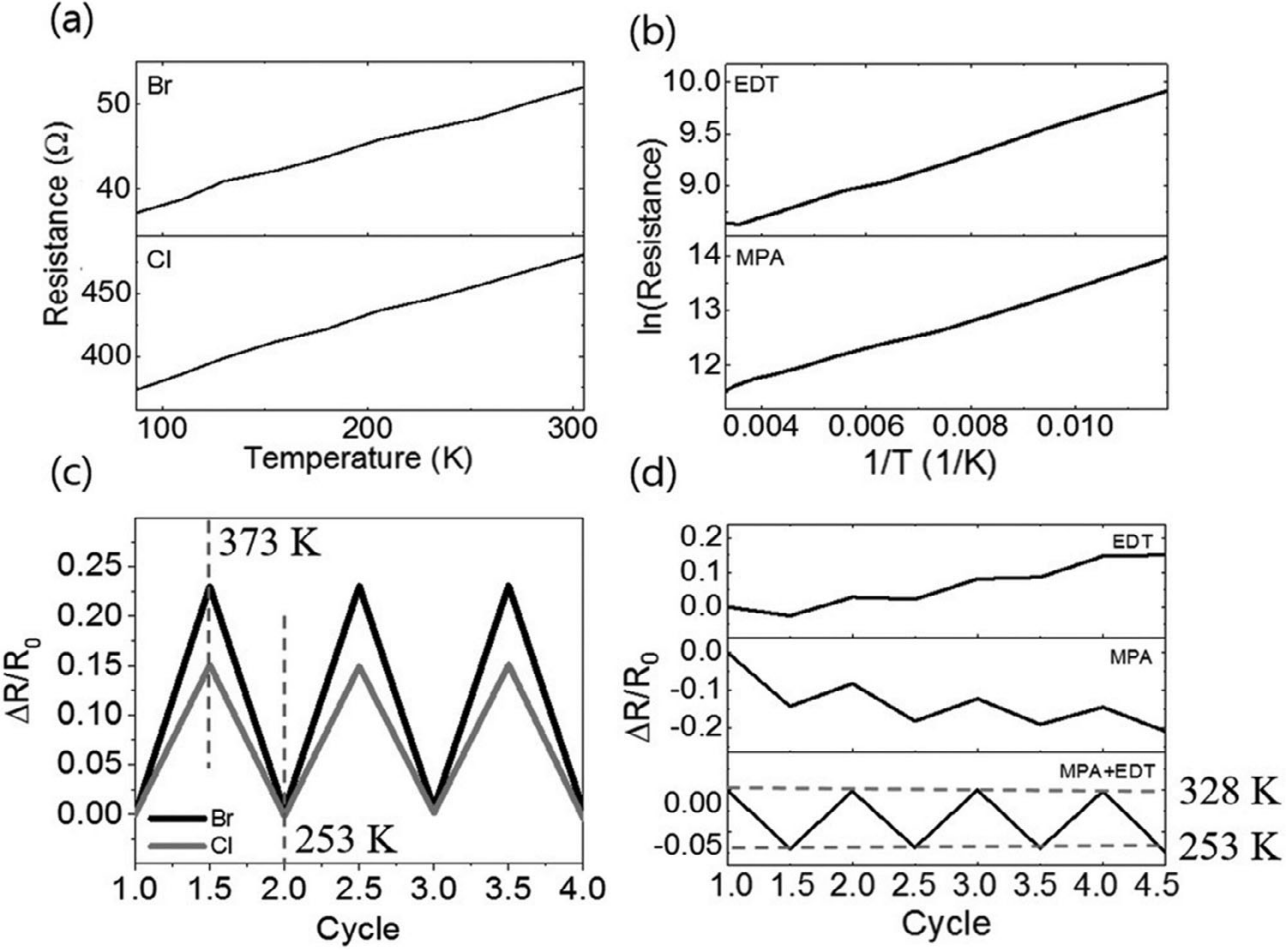
Modification of silver nanocrystals by different ligands (a. inorange ligands, b. orange ligands) leads to a transformation of the transport behavior, and their stability c,d). Reproduced with permission.^[^ ^110^ ^]^ Copyright 2017, Wiley‐VCH.

The thermal resistance effect, one of the most straightforward mechanisms types of temperature sensors, means that as the temperature changes, the sensor's resistance changes correspondingly. Charge transport behaviors about temperature‐dependent have metallic transport and hopping transport. In Figure 12a, metal transport exhibits PTC properties. The free electrons form an electron cloud, so the number of carriers in the metal transport material is constant. When the temperature increases, the probability of collision of carriers with higher energy increases, and then the resistance increases. Usually, it can be expressed in Equation (1):^[^ ^110^ ^]^

(1) $$\frac{\Delta R}{R_{0}}=\alpha \Delta T+G\varepsilon$$

where *α* is the TCR, *G* is the gauge factor, *ε* is the strain. *Gε* is negligible in rigid devices but must be considered in flexible devices.^[^ ^110^ ^]^ In hopping transport, the valence electrons are trapped by the nucleus, and the temperature provides the energy for the valence electrons to escape from the nucleus, causing the valence electrons to become carriers. As the temperature increases, the number of carriers increases and the resistance decreases. Hopping transport fits Arrhenius equation: $R=R_{0}e^{\frac{E_{a}}{kT}}$, where Ea, k is the activation energy for thermally induced hopping and Boltzmann constant, respectively. The transport properties of a material depend on its electronic structure, and a change in the electronic structure may cause a transition from metal transport to hopping transport or hopping transport to metal transport,^[^ ^110^, ^111^ ^]^ in **Figure** **13**.

#### 2.3.2 Seebeck Effect

**Figure 14:**
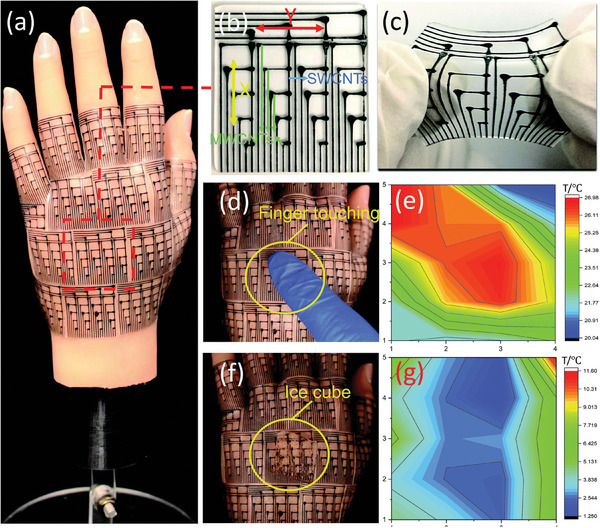
A highly stretchable strain‐insensitive temperature sensor exploits the Seebeck effect, which can detect temperature distribution across the surface of thing. Reproduced with permission.^[^ ^161^ ^]^Copyright 2019, The Royal Society of Chemistry.

The Seebeck effect, a thermal‐electric conversion phenomenon, uses the temperature difference between the two ends of a heterogeneous material to drive carriers into motion and form an electric current (Figure 12b). The Seebeck coefficient (S) is defined as the electric field strength ratio to the temperature gradient: S = dV/dT, where dV and dT are potential differences and temperature differences between the cold end and the hot end. Typically, the Seebeck effect is used as a power generator for energy supply and a temperature measurement device.^[^ ^158^, ^159^ ^]^ Thermocouple temperature measurement in temperature sensors based on the Seebeck effect is a typical successful example, which enables temperature measurement over an ultra‐wide temperature range (−196 −1200 °C).^[^ ^38^ ^]^ In contrast to other temperature measurement mechanisms, the Seebeck effect is not affected by strain, which avoids temperature‐strain signal coupling in flexible stretchable and bendable temperature sensors.^[^ ^160^, ^161^ ^]^ In **Figure** **14**, the double‐walled carbon nanotubes measure the temperature by monitoring the output voltage signal, which does not interfered with strain and can ensure the accuracy of the temperature measurement. The array‐arranged carbon nanotubes also map the temperature distribution. It is also possible to increase the sensitivity of temperature sensors by using different sensitive materials or reducing the thermal conductivity of the substrate.^[^ ^117^, ^159^ ^]^

#### 2.3.3 Pyroelectric Effect

Temperature measurement and power generation using the Seebeck effect is a good choice in spaces with temperature gradient distribution. However, the pyroelectric effect must be chosen in environments without temperature gradients, which is based on the fact that anisotropic materials are spontaneously polarized by temperature changes (dT/dt).^[^ ^158^ ^]^ Pyroelectric materials are polar dielectric materials with a non‐central symmetric structure.^[^ ^162^ ^]^ In Figure 12c, a polar material is spontaneously polarized resulting in a charge of opposite sign at the ends of the material. It is commonly used to fabricate nanogenerators for energy harvesting and conversion and can also be used as self‐powered temperature sensors. When the temperature changes with time, the pyroelectric material generates a current *I*, in Equation (2):^[^ ^163^ ^]^

(2) $$I=\frac{dQ}{dT}=-\mathrm{pA}\frac{dT}{dt}$$

where *Q* is the generated charge, *p* is the pyroelectric coefficient, *A* is the electrode cross section area, and *dT/dt* is the rate of temperature change with time. Extrapolation of Equation (2) yields Equation (3):^[^ ^163^ ^]^

(3) $$T_{2}-T_{1}=-\frac{1}{pA}\int_{t_{1}}^{t_{2}}Idt$$

**Figure 15:**
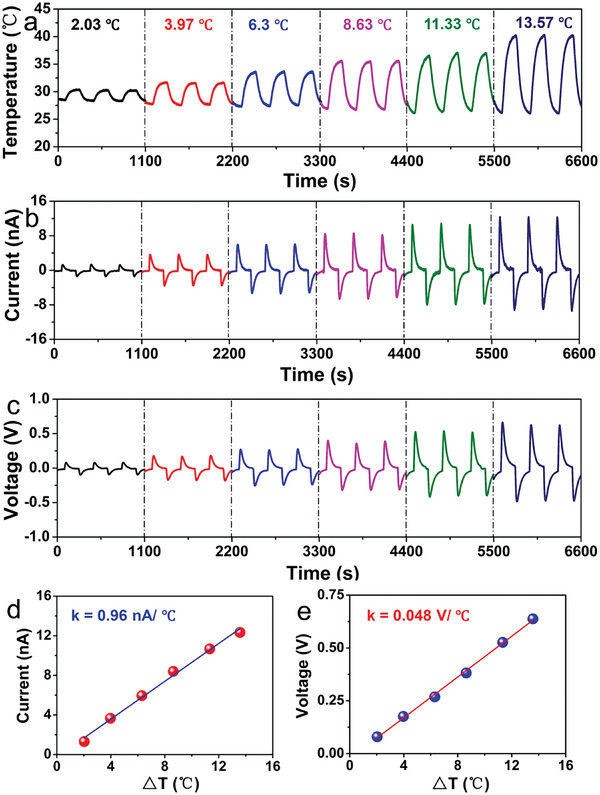
Pyroelectric properties of a single Ag/BTO/Ag sensing device. Reproduced with permission.^[^ ^116^ ^]^ Copyright 2019, Wiley‐VCH.

T_1_ and T_2_ are temperatures at time t_1_ and t_2_, respectively. This means that the final temperature (T_2_) can be obtained by calculating the total current generated over time (t_1_ to t_2_). Many materials with ABO_3_ structure are used as sensitive materials of self‐powder temperature sensors, such as BaTiO_3_, LiNdO_3_, etc.^[^ ^116^, ^162^, ^163^, ^164^ ^]^ For example, Wang and their co‐workers developed a novel 4 × 4 Ag/BaTiO_3_/Ag array sensor with high sensitivity to detect real‐time temperature and pressure changes in **Figure** **15**.

#### 2.3.4 Infrared Radiation

Temperature measurement methods include contact and non‐contact temperature measurement. The methods mentioned above belong to the former, and infrared thermometers belong to the latter. In nature, all objects with a higher temperature than absolute zero (−273.15 °C) constantly radiate energy outward. The total energy radiated and the energy radiated at a particular wavelength are positively correlated with the temperature of the object, the higher the object's temperature, the stronger its ability to send out infrared radiation (Figure 12d). According to the Stefan–Boltzmann law, emitting the total energy radiated (M) of the grey body is proportional to temperature (T) and dependent on the frequency:^[^ ^165^ ^]^ M(T) = CT^n^, where C is a constant, n dependent on the infrared spectral bands. However, radiation detectors receive radiation from the object itself, reflected radiation from the object's surface, and atmospheric radiation. More complex correction formulas are needed to calculate the object's temperature. Infrared thermometry was previously uncommon, but since COVID‐19, infrared thermography has become a necessary temperature‐measuring device in public areas, playing a pivotal role in accurately and quickly identifying individuals with abnormal body temperatures.

Flexible temperature sensors often generate stress in the material during deformation processes such as bending or stretching, and most temperature‐sensitive materials are pressure‐sensitive materials such as CNT, PEDOT, BaTiO_3_, etc. Understanding the temperature measurement mechanism of temperature sensing materials is beneficial to obtain accurate temperature measurement and adjustment performance.

Meanwhile, the reproducibility is one of the important parameters of a sensor, and devices produced in different batches often show a drift in resistance. However, many articles on flexible sensors now characterize the reproducibility of the sensors through cycle testing^[^ ^166^, ^167^, ^168^, ^169^ ^]^ or multi‐batch experiments,^[^ ^170^, ^171^, ^172^ ^]^ and few articles analyze the reasons for the low reproducibility and the methods to improve the experimental reproducibility. Fulvio Michelis et al.^[^ ^171^ ^]^ investigated the changes in the initial resistance of the devices for five different batches of sensors ranging from 3 to 72 devices, with variability ranging from 8.4% to 43%. Uneven deposition of electrode material and variations in ink quality resulted in low device repeatability. Satoko Honda et al.^[^ ^169^ ^]^ prepared a tactile force sensing array based on a textile sheet/conductive silver wire. The sensor showed fluctuations in resistance value after 250000 stable cycles under 5 kPa pressure, and the number of stable cycles decreased with increasing applied pressure. The resistance fluctuations were attributed to the conductive silver wire detaching from the textile sheet. Therefore, we have summarized a few methods to minimize the repeatability problem and the error margin: first, the sensitive material should have good stability; second, the suitable manufacturing method and process should be selected; thirdly, the various types of materials in the device need to be strongly binding in order to ensure the stability of the structure.

### 2.4 Advanced Characterization Methods of Flexible Temperature Sensors

Common methods used to characterize flexible temperature sensors include X‐ray diffraction (XRD), atomic force microscopy (AFM), electron microscopy, and spectroscopy methods such as Raman and infrared/ultraviolet spectroscopy, etc.^[^ ^57^, ^61^ ^]^ These techniques are valuable for a comprehensive understanding of flexible temperature sensors in terms of their crystal structure, surface morphology, local chemistry, and functional properties. Combining multiple techniques allows researchers to obtain a more complete picture of the structure‐property relationships in flexible temperature sensors and, in turn, guides the design of new high‐performance sensors.

Among them, electron microscopy is a powerful tool that enables high‐precision multi‐dimensional studies of materials, regarding their microstructure, chemical composition, and electronic structures, from the micro‐ and nanoscale to the atomic scale. While traditional electron microscopy techniques such as scanning electron microscopy (SEM) and transmission electron microscopy (TEM) are not commonly used for characterizing flexible temperature sensors directly, they can be employed to study the materials and interfaces involved in the sensors. SEM can be used to examine the surface morphology and microstructure of materials used for flexible temperature sensors. TEM provides unique insight into atomic‐scale of interfaces between layers and boundaries between structural domains, leading to an in‐depth understanding of the interactions in materials and their impact on the sensors’ performance. In addition, for flexible temperature sensors incorporated with nanoparticles, TEM holds particular advantages in studying their lattice structure, size distribution, dispersion, and arrangement within the sensor structure. Furthermore, coupled with EDS and EELS systems, both SEM and TEM play a key role in the failure analysis of sensors to identify defects or structural cracks in case of failure or degradation to figure out the way to fabricate high‐performance sensors.

Compared to rigid temperature sensors, flexible temperature sensors require excellent mechanical properties for practical applications, as they are often subjected to continuous stress changes during operation. The variation of stress has important implications for material deformation mechanisms and interfacial contact between the functional material and the substrate/matrix. In addition, the effect of temperature on the interface between the substrate layer and the functional layer cannot be ignored due to the fact that the coefficient of thermal expansion of polymeric materials (10^−5^/K) is at least an order of magnitude larger than that of temperature‐sensitive materials, such as CNTs (10^−6^/K).^[^ ^10^, ^173^ ^]^

Nowadays, microstructural analysis methods for flexible temperature sensors mostly rely on ex situ characterizations, which only provide information about the structure before and after the change, and lacks the characterization of the structural changes during the change. In situ characterizations that visualize the evolution of structure and charge distribution in flexible temperature sensors under the external stimuli of stress and temperature are rarely reported.

**Figure 16:**
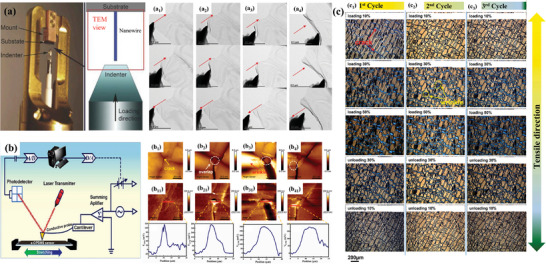
a) In situ TEM tests the changes of graphite flakes under different bending radius, Reproduced with permission.^[^ ^176^ ^]^ Copyright 2019, Elsevier Ltd. b) In situ KPFM reveals the changes of crack surface potential of a‐C film under different tensile strains.^[^ ^175^ ^]^ c) In situ CLSM tests the effect of strain on cracks and wrinkles in a‐C film, Reproduced with permission.^[^ ^175^ ^]^ Copyright 2022, Elsevier B.V.

In‐situ analytical methods based on the study of the effect of mechanical stimuli on the deformation and electrical properties of materials have applications in many materials, the key to which lies in the achievement of material tension, compression, shear, and bending under the test conditions, as well as the quantitative detection of stresses. Li et al.^[^ ^174^ ^]^ systematically introduced in situ TEM based on tensile holders, nanoindentation holders, MEMS devices, thermal bimetallic technology, and their advantages and disadvantages. TEM has the capability of high temporal and spatial resolution to characterize the structure of materials under certain conditions. Flexible temperature sensors in electronic skins and wearable devices undergo repeated deformation, and during deformation, morphology and defects of material are characterized by in situ TEM and HRTEM to guide the preparation of highly flexible temperature sensors (**Figure** **16**a). In situ Kelvin probe force microscopy (KPFM) can characterize the material morphology and the potential difference of surface contact potentials (Figure 16b).^[^ ^175^ ^]^ In situ Raman and in situ IR/UV spectroscopy can reveal changes in the structure of temperature sensors in terms of chemical bonding.^[^ ^175^, ^176^ ^]^ Confocal laser scanning microscopy (CLSM) has been used to characterize the surface morphology of materials (Figure 16c).^[^ ^175^ ^]^ In situ characterization methods based on temperature variations require the fittings of heating elements. In situ XRD,^[^ ^177^ ^]^ in situ atomic force microscope (AFM),^[^ ^178^ ^]^ in situ Raman^[^ ^179^, ^180^ ^]^ can be used to analyze the crystal structure, surface morphology, and carrier distribution of materials, as well as changes in bonding. These in situ characterization methods provide a new insight into the mechanisms of variation in the mechanical and electrical properties of flexible temperature sensors.

## 3 Manufacturing Technology

With the development of science and technology and the increased application scenarios of flexible temperature sensors, the research and preparation of flexible temperature sensors have put forward higher requirements. Flexible, stretchable, lightweight, ultra‐thin, and transparent flexible temperature sensors have attracted considerable attention. In order to face the high demands placed on the devices by the market, the preparation process of the devices is facing enormous challenges. Depending on the state of the processed material, the preparation methods of flexible electronic devices can be broadly classified into three categories: solid‐state processing, solution processing, and vapor‐phase deposition. This section will focus on the processing methods for flexible devices and their application in preparing flexible temperature sensors.

### 3.1 Solid‐State Processing

**Figure 17:**
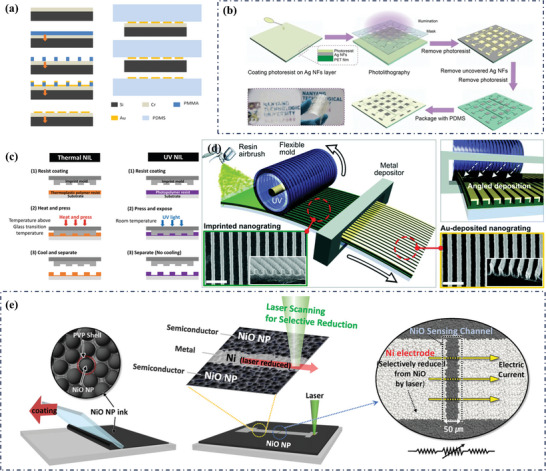
a) Schematic diagram of photolithography, Reproduced with permission.^[^ ^186^ ^]^ Copyright 2022, MDPI. b) Flow chart of the photolithography process to fabricate the patterned Ag NFs electrode and to package as the single electrode triboelectric nanogenerator (TENG) sensor array device,^[^ ^182^ ^]^ Copyright 2021, MDPI. c) Two working principles of nanoimprinting lithography,^[^ ^187^ ^]^ d) 3D structures prepared based on roll‐to‐roll nanoimprinting. Reproduced with permission.^[^ ^185^ ^]^ Copyright 2013, The Royal Society of Chemistry. e) Laser direct writing prepared Ni‐NiO‐Ni temperature sensors. Reproduced with permission.^[^ ^35^ ^]^ Copyright 2019, Wiley‐VCH.

Photolithography is a high‐precision semiconductor processing technology vital in micro‐ and nano‐fabrication technologies. It forms high‐precision and small‐linewidth patterns on a substrate using an optical system, a photoresist, and a mask plate. It is now a key technology for preparing highly integrated, small‐size chips. Its working process contains several exemplary steps: spin‐coating photoresist, exposure, etching (deposition), and cleaning, as shown in **Figure** **17**a. Photolithography is typically used for patterning on rigid substrates. However, with the increased demand for processing flexible materials, photolithography can also be used to process flexible devices (Figure 17b), such as flexible gas sensors and haptic sensors.^[^ ^181^, ^182^, ^183^, ^184^ ^]^ Although photolithography is not limited by the hardness of the material to be processed and is capable of processing delicate device structures, photolithography is expensive, energy‐intensive, and wasteful of materials, and the preparation process is cumbersome, which has led researchers to explore alternatives to photolithography. In the 1990s, nanoimprinting was invented, and after 30 years of development, it has made it possible to replace photolithography. Nanoimprint lithography enables the patterning of materials on a substrate by contacting the processed material with a stencil, then curing by heating or UV irradiation, and finally, molding (Figure 17c). This technology is simple and less energy‐intensive than photolithography and facilitates the shaping of 3D structures directly on the substrate (Figure 17d).^[^ ^185^ ^]^ For nanoimprint lithography to become an affordable alternative to conventional lithography, it must also overcome low precision and yield problems. In addition to the methods mentioned above, laser direct writing technology has also shone in the design of chip patterns. Laser direct technology does not require templates; the pattern can be entered directly into the computer, and the program controls the laser to hit the substrate and write the pattern directly. This method again simplifies the process, and it, through a unique design, can achieve a seamless connection between the sensitive material and the electrode to regulate the performance of the electronic components (Figure 17e).^[^ ^35^ ^]^

### 3.2 Liquid Phase Processing

**Figure 18:**
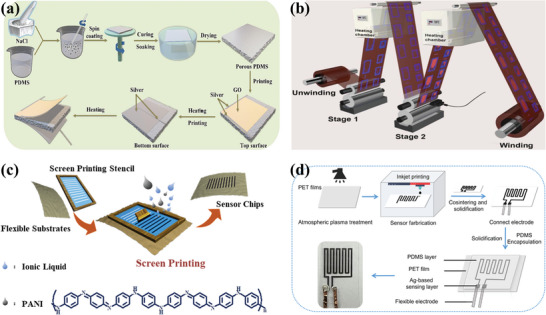
Four processes for preparing flexible temperature sensors, a) spin‐coating, Reproduced with permission.^[^ ^188^ ^]^ Copyright 2023, Elsevier B.V., b) roll‐to‐roll,^[^ ^196^ ^]^ Copyright 2019, IEEE., c)screen printing, Reproduced with permission.^[^ ^189^ ^]^ Copyright 2021, Elsevier Ltd., and d) inkjet printing, Reproduced with permission.^[^ ^190^ ^]^ Copyright 2023, IOP Publishing Ltd.

The liquid phase processing process refers to the formulation of materials containing specific functions into a paste, and then through some processing methods, such as screen printing, roll‐to‐roll processing, spin‐coating, inkjet printing, and other processes to print the paste on the substrate into a design pattern (**Figure** **18**). The key to the liquid‐phase process is the performance of the paste. The rheology and stability of the slurry are influenced by many parameters: viscosity, contact angle, zeta potential, particle size, content, and shape of the functional phase. In addition, different processing methods require different properties from the slurry.

**Figure 19:**
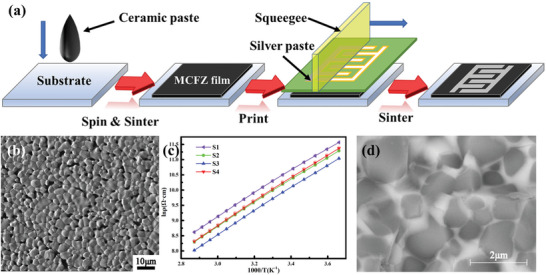
Spin‐coating metal oxide paste to prepare temperature sensors. a) The process diagram of spin‐coating metal oxide pastes and screen‐printing silver electrodes, b) SEM, and c) the lnρ−1000/T curve of ceramic thick films formed by spin‐coated high estimated content ceramic pastes. Reproduced with permission.^[^ ^191^ ^]^ Copyright 2021, Elsevier B.V. d) The glass phase fills in the interstices of the ceramic thick film to form a dense ceramic. Reproduced with permission.^[^ ^192^ ^]^ Copyright 2022, Elsevier B.V.

With its low cost and good compatibility with the substrate, the spin‐coating process is an important step in preparing electronic devices. A certain amount of paste is dropped on the substrate, and the centrifugal force generated by the spin of the substrate is used to uniformly coat the paste on the substrate to form a film‐like device.^[^ ^188^ ^]^ Changing the viscosity of the paste and the substrate spin speed can achieve the control of film thickness. Huo et al. prepared temperature sensors by spin‐coating polymetallic oxide pastes (**Figure** **19**) to achieve temperature measurements in the range of 0–75 °C. They compared the effects of different functional phase contents on the morphology and electrical properties of thick‐film temperature sensors.^[^ ^191^ ^]^ As the functional phase content increases, the resistivity first decreases and then increases due to cracking. The cracking of samples with high solid content can be improved by doping the glassy phase.^[^ ^192^ ^]^ However, forming patterned electronics on a substrate cannot be achieved using spin‐coating alone; it requires other preparation processes.

Screen printing is a traditional technique in which a functional ink or paste is dripped onto a screen, and then a squeegee is used to push the paste into a grid on the screen to form a predetermined pattern on the substrate.^[^ ^193^ ^]^ The adjustment of the pastes focuses on viscosity and thixotropy: low‐viscosity inks lead to poor printed pattern quality, and high‐viscosity inks bring about poor surface flatness. Highly thixotropic inks facilitate smooth passage through the screen mesh and maintain their printed shape. The accuracy of screen printing is affected by many factors, including the nature of the paste, the screen, the substrate, and the squeegee coating process, all of which affect the resolution. Nevertheless, screen printing technology, with the advantages of low cost, high manufacturing efficiency, and large‐area preparation, is widely used in the processing of electronic devices. Screen printing has also been used for the preparation of flexible temperature sensors. Liu and their co‐workers screen‐printed indium oxide/indium tin oxide pastes on PI substrates to prepare flexible temperature sensors, and the sensitivity of the thermoelectric effect‐based temperature sensors could reach up to 162.5 µV °C^−1^.^[^ ^194^ ^]^ Florian Le Goupil et al. prepared high‐precision (0.01 K) flexible temperature sensors using an all‐printing process.^[^ ^195^ ^]^ Roll‐to‐roll technology is a print technology that enables multiple prints, large‐area preparation, and integrated printing/drying, making it possible to take laboratory electronics to large‐scale commercial applications. Roll‐to‐roll technology enables high‐volume, high‐quality printing of electronics. For example, it has been used to implement intelligent food labeling to replace the existing “Use‐By” date system.^[^ ^196^ ^]^

**Figure 20:**
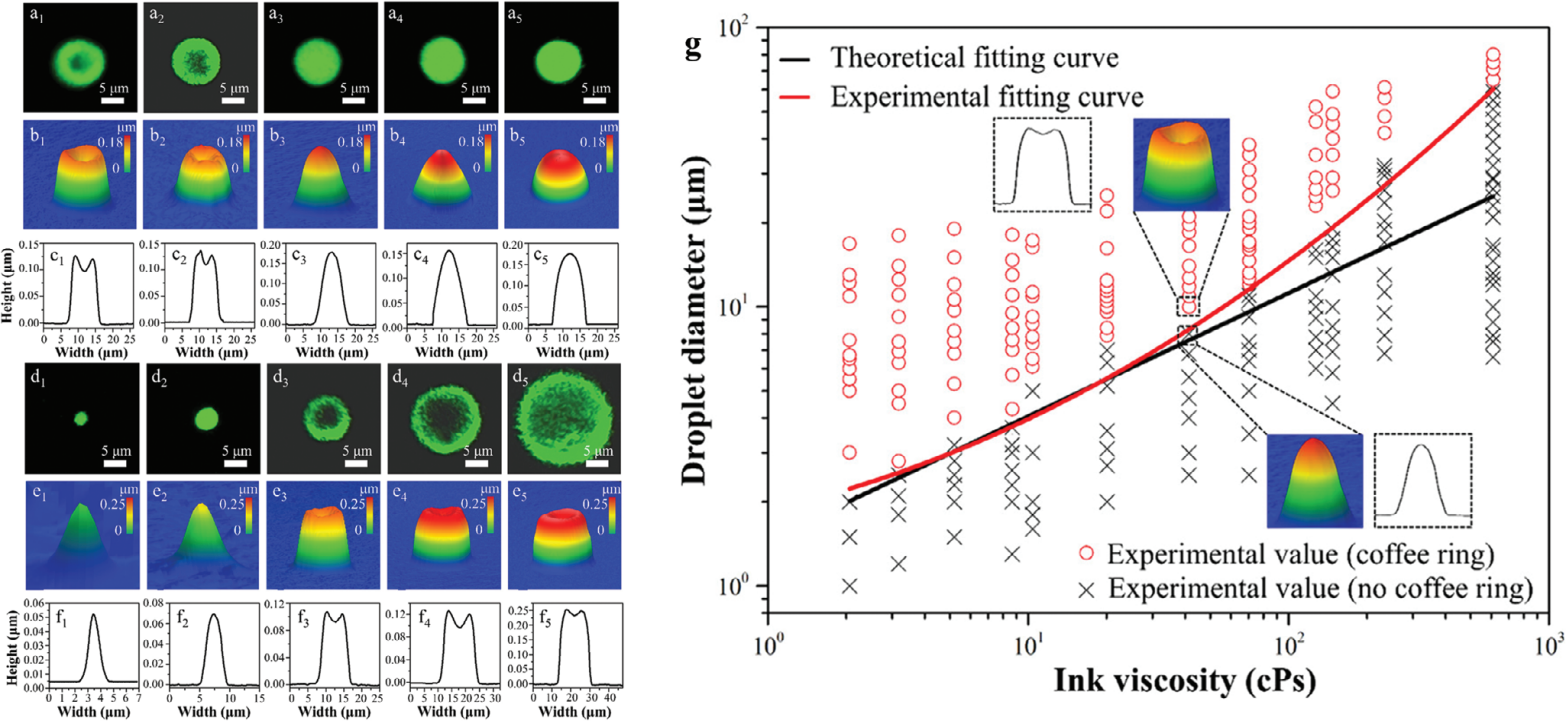
Morphologies of EHD‐printed dots with a–c)the same size (10 µm) and different viscosities, d–f) the same viscosities (10.39 cPs) and different size. Plot depicting the correlation of droplet morphology with solution viscosity and droplet size. Reproduced with permission.^[^ ^202^ ^]^ Copyright 2022, American Chemical Society.

Screen printing and roll‐to‐roll technology share the same feature: ink directly passes through the screen or engraved rollers to form a predetermined pattern on the substrate. A printing technique with this feature is called contact printing. While both methods offer excellent cost efficiencies, attention must be paid to the potential for sample contamination during repeated print sessions. In non‐contact printing, no template and a computer‐controlled nozzle jets ink onto the surface of the substrate to form a specific pattern.^[^ ^197^ ^]^ Inkjet printing is known as non‐contact printing. The effectiveness of inkjet printing depends on the quality of the ink and the nature of the ink can be expressed using the Ohnesorge value (Oh), as in Equation (4):^[^ ^198^ ^]^

(4) $$Oh=\frac{\sqrt{We}}{Re}=\frac{\eta }{\sqrt{\gamma \rho a}}$$

where η, γ, and ρ are the viscosity, surface tension, and density of the ink, respectively, and a is the characteristic size of the nozzle. The ink has high printability when 1/Oh is located at 1–10. Moreover, the stability of the paste was characterized by a static settling experiment; the smaller the settling rate, the more stable the ink. During the drying process of inkjet‐printed droplets on the substrate, the solvent in the contact line between the droplet and the substrate evaporates first, and the solid particles in the center of the droplet follow the solvent toward the contact line, which results in the formation of a ring‐like deposit on the substrate.^[^ ^199^ ^]^ This phenomenon is not conducive to the formation of a uniform film. Zhang et al.^[^ ^200^ ^]^ demonstrated that ring deposition can be effectively inhibited by increasing the viscosity or decreasing the print size (**Figure** **20**a–f). It concluded that the competition between the two factors, solvent evaporation, and particle diffusion, determines the shape of the deposition. The threshold size and viscosity for the formation of ring deposition were obtained through theoretical calculations, and the calculation curves and experimental curves were in high consistency (Figure 20g).

Inkjet printing is one of the best methods to prepare microdevices. It is possible to print metal inks,^[^ ^190^ ^]^ carbon nano‐inks, conductive polymer inks,^[^ ^201^ ^]^ and metal oxide inks^[^ ^36^ ^]^ on substrates to form patterns with small line widths, facilitating miniaturization and device integration. Tomohiko^[^ ^36^ ^]^ used inkjet printing to prepare ultra‐light and ultra‐thin flexible temperature sensors with a thickness of only 5um and a mass of only 21 mg. They used ultra‐light flexible PI material as a substrate and used a laser to sinter the temperature‐sensitive material. The lightweight material is favorable for applying flexible temperature sensors in wearable devices.

**Figure 21:**
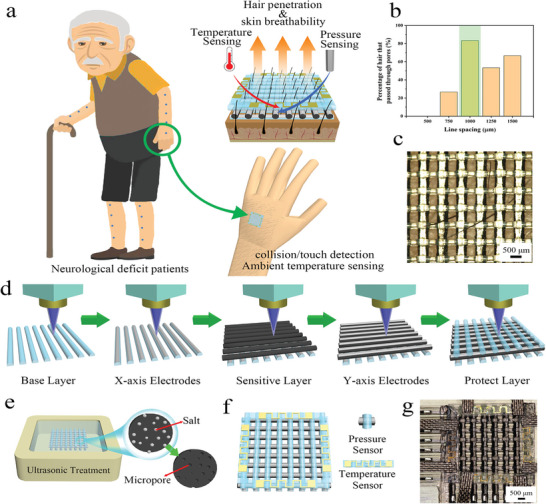
3D printed bimodal breathable electronic skin. Reproduced with permission.^[^ ^205^ ^]^ Copyright 2022, American Chemical Society.

3D printing, also known as additive manufacturing, is a preparation technique for achieving complex 3D structures through layer‐by‐layer printing, which has brought about the technological revolution in many fields by its flexibility, efficiency, and low cost.^[^ ^203^ ^]^ Typically, 3D printing consists of four steps: creation of a 3D model, acquisition of a layer‐by‐layer pattern, printing of the layer‐by‐layer pattern, and curing.^[^ ^204^ ^]^ Compared to the other liquid‐phase processes mentioned above, 3D printing has more strict requirements for ink formulations and printing solutions – print smoothness, shape retention, and high resolution. Thanks to the characteristics of 3D printing, the process of preparing flexible wearable electronics with special structures is simplified. Considering the comfort and safety of the human body, achieving breathability in wearable devices has received a lot of attention in recent years. However, it is very difficult to achieve breathability in devices with conventional top and bottom structures. Direct shaping of mesh structures with the help of 3D printing offers a possibility to achieve breathability of devices,^[^ ^145^, ^205^ ^]^ as shown in **Figure** **21**.

The advantages and disadvantages of the five liquid‐phase processing processes described above are compared in **Table** **5**. Liquid‐phase processing methods are more suitable for preparing flexible electronic devices. Liquid‐phase processing has more cost advantages, shorter processing time, and less material waste than solid‐phase processing.

**Table 5:** Comparison of the advantages and disadvantages of five molding methods.

Method	Characteristic	
	Advantages	Disadvantages
Spin coating	Simple process, low equipment cost, low ink requirements	Patterning cannot be achieved independently
Screen printing	Low cost and large processing area for commercial production	Contact printing, samples are susceptible to contamination
Roll‐to‐Roll		
Inkjet prinking	No need for templates, flexible print patterns	High ink requirements and high printing costs
3D prinking	Preparing complex 3D structures, simple process, low cost	High ink and requirements

### 3.3 Vapor Phase Deposition

**Figure 22:**
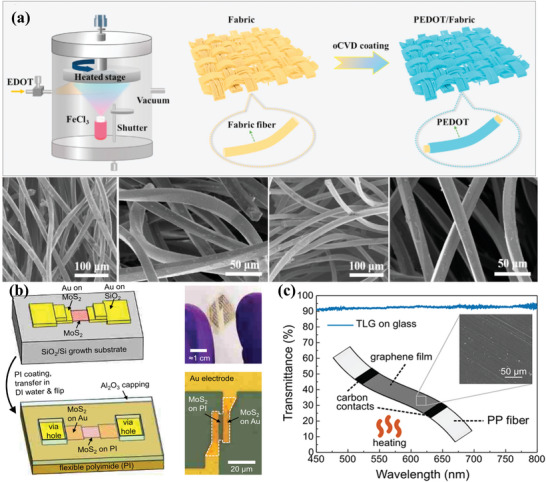
Chemical vapor deposition schematic and applications. a) Preparing PEDOT/fabric composite by CVD. Reproduced with permission.^[^ ^206^ ^]^ Copyright 2022, Elsevier Ltd. b) Structure of molybdenum disulphide flexible temperature sensors. Reproduced with permission.^[^ ^37^ ^]^ Copyright 2022, American Chemical Society. c) Transmittance as a function of wavelength for trilayer graphene on glass with an SEM image of graphene‐coated fibers. Reproduced with permission.^[^ ^209^ ^]^ Copyright 2020, American Chemical Society.

The vapor phase deposition method is a thin film preparation method in which the reactants are vaporized by heating, plasma bombardment, UV irradiation, and other technological methods and then diffused onto the substrate surface to deposit a film. This method has no solvent involvement and offers significant advantages in dealing with insoluble polymers and solvent‐sensitive substrates.^[^ ^206^ ^]^ Depending on whether or not they react on the substrate surface, the vapor phase deposition methods can be classified into physical vapor phase deposition (PVD) and chemical vapor phase deposition (CVD). Magnetron sputtering is a common physical vapor deposition method, which typically sputters metallic materials such as silver and platinum onto flexible substrates to prepare flexible metal‐based temperature sensors.^[^ ^207^, ^208^ ^]^ The intrinsic ductility of metallic materials makes metal‐based flexible temperature sensors excellent for comfort and flexibility. However, the small temperature coefficient of resistance limits the practical application of the devices. Considering the need for highly sensitive flexible temperature sensors temperature for some specific applications, metal oxide‐based temperature sensors with high temperature sensitivity have been prepared by magnetron sputtering.^[^ ^112^ ^]^ The metal oxide targets used in this process must be customized, and the preparation of ceramic targets must overcome many difficulties (shaping, cracking). Graphene and metal sulfides have been widely used in flexible temperature sensors due to their high carrier density and excellent flexibility.^[^ ^37^, ^61^, ^62^, ^117^ ^]^ The conventional methods for preparing graphene and metal sulfides are mechanical exfoliation and melt‐annealing, respectively, which could be more efficient in terms of productivity and complicated processes. Compared to other methods, chemical vapor deposition is a promising method for preparing thin film materials because of the high quality and low cost of the prepared materials (**Figure** **22**a).^[^ ^209^ ^]^ The flexible single‐layer molybdenum disulfide temperature sensor prepared by chemical vapor deposition can quickly detect temperature changes within a few microseconds, and its response speed exceeds that of most flexible temperature sensors, making it an excellent candidate for many applications (Figure 22b).^[^ ^37^ ^]^ The CVD‐prepared carbon‐paste‐graphene composite makes the flexible temperature sensor highly bendable and washable (Figure 22c).^[^ ^209^ ^]^

## 4 Applications

Flexible temperature sensors have different needs in different application situations, and the following is an introduction to the application of flexible temperature sensors from both human and physical aspects.

### 4.1 Flexible Temperature Sensors for the Human Body

**Figure 23:**
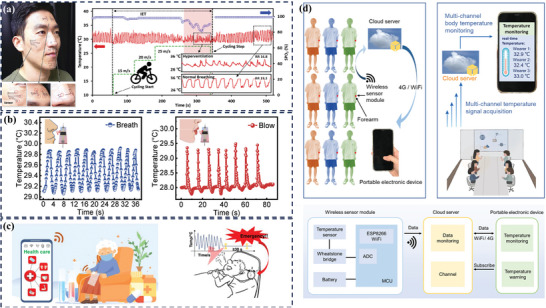
a) Body‐temperature detection. Reproduced with permission.^[^ ^35^ ^]^ Copyright 2019, Wiley‐VCH. b) Breathing rate and blowing detection. Reproduced with permission.^[^ ^62^ ^]^ Copyright 2021, Elsevier Ltd. All rights reserved. c) Health monitoring and disease prevention. Reproduced with permission.^[^ ^38^ ^]^ Copyright 2022, IOP. d) High throughput wireless body temperature monitoring system. Reproduced with permission.^[^ ^61^ ^]^ Copyright 2022, Wiley‐VCH.

Flexible, highly sensitive, and fast‐responding temperature sensors are used in the human body mainly to monitor body temperature and breathing rate. Body temperature is closely related to human health and is an outward sign of cellular synergy in the body. As shown in **Figure** **23**a, a flexible temperature sensor can be placed on the skin to measure the temperature directly. Flexible temperature sensors provide more accurate and real‐time temperature monitoring than traditional rigid temperature sensors. The normal temperature range for each part of the body is different, and the temperature in the armpit is usually tested to be 36–37.3 °C. Prediction of early disease through body temperature is an urgent need in preventive medicine. Body temperature monitoring with flexible temperature sensors is mainly used for health monitoring of infants and the elderly (Figure 23c), monitoring of wound healing after surgery, and monitoring of mental health. On the other hand, in addition to body temperature, the monitoring of breathing rate can be achieved by analyzing the difference in temperature between inhalation and exhalation (Figure 23b).^[^ ^61^, ^62^ ^]^ The breathing rate represents the change of metabolic demand in the body, for example, the exercise breathing rate is 2–3 times of the resting breathing rate. Nowadays, many people's bodies are in a sub‐healthy state, sudden death, heart attack/brain attack, apnoea syndrome patients are getting younger, and the golden time for rescue is only a few minutes. Monitoring the breathing rate and connecting to the alarm system is very important for timely detection of the condition and saving lives.

Therefore, a great deal of work has been done to achieve the fictionalization of flexible temperature sensing using the above as an application guide for flexible temperature sensors. For temperature measurement, it is important to achieve the properties of fast response, high sensitivity, and high accuracy of flexible temperature sensors. Considering the portability, comfort, and aesthetics of wearing, wireless (Figure 23d), breathable and invisible flexible temperature sensors have been prepared.

### 4.2 Flexible Temperature Sensors for Objects

In addition to being used for human physiological temperature detection, flexible temperature sensors are also used for temperature measurement and monitoring of robot skins, prosthetic limbs, wearable devices, and energy storage batteries. In contrast to the former, flexible temperature sensing for objects does not require comfort or aesthetics, and the key to the sensor's application lies in its electrical and mechanical properties.

**Figure 24:**
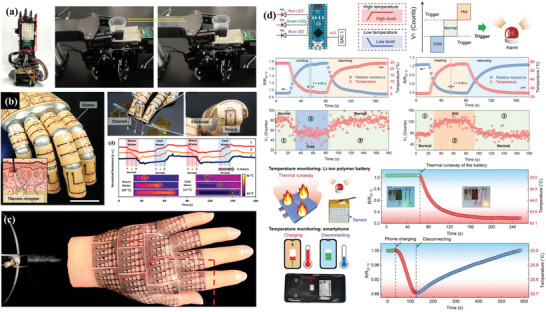
a) Wearable device with mechanical dexterous hands for large‐area temperature detection. Reproduced with permission.^[^ ^38^ ^]^ Copyright 2022, IOP. b) Robotic fingers for determining the direction of liquid flow. Reproduced with permission.^[^ ^35^ ^]^ Copyright 2019, Wiley‐VCH. c) Detecting temperature distribution across the surface of a soft robotic arm,^[^ ^161^ ^]^ Copyright 2019, The Royal Society of Chemistry. d) Applications for the flexible temperature sensors in temperature monitoring and warning system. Reproduced with permission.^[^ ^210^ ^]^ Copyright 2024, Wiley‐VCH.

The integration of flexible temperature sensors in manipulators enables the measurement of the temperature of the gripped object, the determination of the direction of fluid flow, and the measurement of the temperature distribution on flat or curved surfaces (**Figure** **24**a–c).^[^ ^35^, ^38^, ^161^ ^]^ The application of flexible temperature sensors in machine skins has positive significance for automated control of production in the industrial fields.

The number of new energy vehicles has surged in the last decade. As the energy density of storage batteries has been increased generation by generation to meet the demand of users for long mileage, the safety of batteries has become one of the biggest issues for the public. Battery thermal out of control is considered to be the main cause of fire accidents during the charging and discharging of batteries. Lu et al. prepared NiO/CNTF flexible temperature sensors with outstanding deformability and ultra‐high sensitivity (−20.2% °C^−1^), and designed a temperature detection system for temperature management of energy storage devices (Figure 24d).^[^ ^210^ ^]^ By installing a flexible temperature sensor on the surface of the battery, when thermal out of control occurs in the battery, the temperature will rapidly increase by two to three times within 1 min, enabling monitoring and prevention of potential fire hazards caused by overheating of the battery. This temperature measurement system is not only applicable to the monitoring of car batteries but is also effective for charging other electronic devices, such as mobile phones, computers, and electric vehicles.

Textile‐based wearable flexible temperature sensors have high applications in healthcare and smart clothing. Textile technology is utilized to weave sensitive materials into arbitrary wearable devices such as gloves, smart clothes, etc.^[^ ^211^, ^212^ ^]^

## 5 Conclusion and Future Prospects

Along with excellent properties such as flexibility, bendability, and conformability, flexible temperature sensors can be used in electronics, energy, and healthcare. Without hesitation, the successful application of flexible temperature sensors will contribute to social progress and industrial reform. This article introduces the material, sensing mechanism, preparation method, and applications of flexible temperature sensors. There is an increasing variety of temperature‐sensitive materials for flexible temperature sensors, including carbon nanomaterials, composite polymer materials, metal materials, and other inorganic materials. Different materials enable flexible temperature sensors to obtain excellent properties, such as high sensitivity, high flexibility, fast response/recovery times, etc. Selection of suitable preparation methods can simplify the process and save time and preparation costs.

Although many people have made outstanding contributions in this field, there is an enormous amount of work to be done: first, performance optimization. Most of the research on the performance of flexible temperature sensors has been focused on sensitivity, stretch‐ability, and faster response time, while there is less research on other essential properties, such as air permeability, mass dimension, and transparency. Second, multi‐functionality. Skin or tactile senses can sense temperature changes and other signals, such as pressure, humidity, etc. Besides, some functions that skin does not have can be integrated into a device to achieve multi‐functionality. Third, miniaturization. Since the history of electronic products, integration, and miniaturization have been significant trends. In addition, some miniature devices require small sensors, such as surgical robots that need to enter the human body by blood vessels. Fourth, optimization of circuitry. Portable devices should achieve low power consumption to prolong the life of the device, as well as wireless transmission of signals to receive signals in various electronic devices such as mobile phones, smartwatches, and other mobile terminals for real‐time monitoring. A technology from an idea takes a long time to be commercially applied. Overall, flexible temperature sensors will bring many benefits to everyday life and industry, and we are optimistic that their widespread use will have unlimited possibilities to expand how things perceive temperature.

## Conflict of Interest

The authors declare no conflict of interest.
